# Enhanced metabolic process to indole alkaloids in *Clematis terniflora* DC. after exposure to high level of UV-B irradiation followed by the dark

**DOI:** 10.1186/s12870-016-0920-3

**Published:** 2016-10-24

**Authors:** Cuixia Gao, Bingxian Yang, Dandan Zhang, Meng Chen, Jingkui Tian

**Affiliations:** 1 Institute of Biomedical Engineering, College of Biomedical Engineering & Instrument Science, Zhejiang University, Zheda Road 38, Hangzhou, 310027 China; 2 Ministry of Education Key Laboratory for Biomedical Engineering, Zhejiang University, Hangzhou, China

**Keywords:** Ultraviolet-B irradiation, *Clematis terniflora* DC., Metabolomics, Proteomics, Amino acid metabolism, Indole alkaloid biosynthesis

## Abstract

**Background:**

Indole alkaloids, which characteristically contain an indole nucleus, have pharmaceutical potential in a diverse range of applications. UV-B can elicit the accumulation of indole alkaloids. The indole alkaloid (6-hydroxyl-1H-indol-3-yl) carboxylic acid methyl ester with cytotoxic activity was found to accumulate in *Clematis terniflora* DC. leaves after exposure to high level of UV-B irradiation and the dark. However, a more in-depth analysis of the process behind this response has not yet been performed. Therefore, an integrated approach involving metabolomic, proteomic, and transcriptomic analyses is essential to detail the biosynthetic mechanisms of the regulation of indole alkaloid under binary stress.

**Results:**

Indole alkaloid (6-hydroxyl-1H-indol-3-yl) carboxylic acid methyl ester was found to increase 7-fold in *C. terniflora* leaves post-treatment with high level of UV-B irradiation followed by an incubation in the dark compared with pre-treatment. Analysis by proteomics and metabolomics indicates a decrease in photosynthesis and carbohydrate metabolism, respectively. By contrast, amino acid metabolism was activated by this binary stress, and, specifically, the genes involved in the metabolic pathway converting shikimate to L-tryptophan were concurrently upregulated. Metabolites involved in indole biosynthesis (shikimate metabolic) pathway were anthranilate, indole, and L-tryptophan, which increased 2-, 441-, and 1-fold, respectively. In addition, there was an increase of 2- and 9-fold in L-serine deaminase (L-SD) and L-tryptophan synthase activity in *C. terniflora* leaves after exposure to high level of UV-B irradiation and the dark.

**Conclusions:**

(6-hydroxyl-1H-indol-3-yl) carboxylic acid methyl ester was found to increase in response to high level of UV-B irradiation followed by an incubation in the dark, implying that indole alkaloid biosynthesis was activated in *C. terniflora* leaves. Analysis of perturbations in metabolism in these leaves demonstrated that amino acid metabolism was specifically activated by this binary stress. In addition, an enhancement in serine level and L-SD activity was noted, which likely leads to an accumulation of pyruvate that, in turn, supplies shikimate metabolic pathway. The genes, metabolites, and L-tryptophan synthase activity that are involved in the metabolic pathway leading from shikimate to L-tryptophan all increased under the experimental binary stress, resulting in an enhancement of indole biosynthesis (shikimate metabolic) pathway. Therefore, the metabolic process to indole alkaloids in *C. terniflora* was enhanced after exposure to high level of UV-B irradiation followed by the dark.

**Electronic supplementary material:**

The online version of this article (doi:10.1186/s12870-016-0920-3) contains supplementary material, which is available to authorized users.

## Background

Indole alkaloids, which characteristically contain an indole nucleus, have pharmaceutical potential in a diverse range of applications, including as cytotoxic [1, 2], antiviral [3], anti-malarial [3], anti-inflammatory [4], and anti-cancer [5] agents. Although the indole nucleus is a nearly ubiquitous component of biologically active natural products, typically only low amounts of indole alkaloids are found in plants. One exception is *Catharanthus roseus*, which produces high amounts of indole alkaloids and is the sole source for the commercial production of several of these compounds [6]. Studies have been performed on increasing the production of indole alkaloids in cell culture [7, 8]. The indole nucleus is an important heterocyclic system that is derived from shikimate metabolic pathway, and provides the framework for indole alkaloids. The incorporation of an indole nucleus, a biologically accepted pharmacophore in indole alkaloids, results in a versatile heterocyclic structure that has a wide spectrum of biological activities [9].

Ultraviolet-B (UV-B) irradiation has biological impact through the modification of the architecture and structure of plants, and has combined effects in damage, repair, and acclimation [10–12]. For example, UV-B irradiation has obvious effects on the accumulation on endogenous secondary metabolites in plants [13], which is likely a response by the plant response to this stimulus. A large range of metabolites with pharmaceutical potential has been investigated through a number of studies, including indole alkaloids in *Catharanthus roseus* [7, 14], caffeoylquinic acid and iridoids such as secologanic acid, secoxyloganin, secologanin, and (E)-aldosecologanin in *Lonicera japonica Thunb.* [15], and diels-alder adduct in *Morus alba* L*.* [16], which accumulated after exposures to high level of UV-B irradiation followed by the dark. Previous research has revealed that UV-B is an elicitor of indole alkaloids accumulation [17, 18]. Specifically, an accumulation of ajmalicine, vindoline, catharanthine, and strictosidine from alkaloids biosynthesis through enhancement of tricarboxylic acid cycle was uncovered [14]. However, the process behind this biological response that results in indole alkaloids accumulation after exposure to high level of UV-B irradiation and the dark has not yet been characterized.

To investigate this, an integrated approach involving metabolomic, proteomic and transcriptomic analyses is essential to detail the biosynthetic mechanisms of the regulation of indole alkaloids, particularly of indole nucleus. Recently, proteomics and metabolomics have been widely applied to characterize the mechanisms by which plants respond to UV-B stress. Proteomic analysis indicated that indole alkaloids in *Catharanthus roseus* accumulated in a manner related to the increase of tricarboxylic acid cycle associated proteins and the decrease of photosynthesis related proteins following high level of UV-B irradiation and the dark [14]. Metabolomic analysis uncovered an increase in flavonoids, anthocyanins, and polyphenols, and a decrease in phenolic precursors in *Populus x canescens* during the first 36 h of UV-B treatment [19]. In addition, proteomics and metabolomics were used to investigate the reactive oxygen species scavenging system and γ-aminobutyric acid shunt pathway activated by high level UV-B irradiation and the dark in leaves of *C. terniflora* [20].

To explore the mechanisms by which indole biosynthesis responds to high level of UV-B irradiation and the dark, *Clematis terniflora* DC. was used as the model organism. *C. terniflora* belongs to *Ranunclaceae*, and is widely spread in Asia (especially eastern regions of China, i.e. Zhejiang Province), Europe, and Africa [21, 22]. Historically, it has long been used as folk medicine in China with important pharmaceutical properties, including anti-inflammatory [23, 24], anti-nociceptive, and antipyretic [25] activities. Furthermore, a novel indole alkaloid (6-hydroxyl-1H-indol-3-yl) carboxylic acid methyl ester with cytotoxic activity against human ECA-109 cell was previously isolated from *C. terniflora* [1], supplying us with an opportunity to investigate indole biosynthesis in this organism for the first time.

## Methods

### Plant materials and treatments

*Clematis terniflora* DC. seeds were incubated in water until germination and then sown into seedbeds. The seedlings were then transplanted into potted containers and placed in a greenhouse with a constant temperature of 28–30 °C, relative humidity of 70–80 %, and white light irradiance of 160 μmol m^-2^ s^-1^. After 6 weeks, the plants were exposed to UV-B irradiation in a cabinet with a controlled temperature of 25–30 °C, and relative humidity of 80 %. The intensity of the UV-B irradiation on the surface was 120.8 μW cm^-2^ as determined using a UV Light Meter (Beijing Normal University, Beijing, China), which is considered a high level of UV-B irradiation. After being irradiated by UV-B for 5 h, the plants were incubated in the dark for 36 h. Plants irradiated with light (irradiance of 160 μmol m^-2^ s^-1^) were used as controls. Leaves located in the lowest 10–60 cm of each plant were collected. For metabolomic and proteomic analyses, 3 independent biological replicates were used.

### Metabolite extraction and analysis by high performance liquid chromatography time-of-flight mass spectrometry

A portion (300 mg) of each freeze-dried sample was added to 3 mL of methanol and the samples were sonicated for 1 h at 4 °C. The suspension was centrifuged at 10,000 × g for 10 min at 4 °C and the supernatant was filtered through a syringe-driven filter (0.45 μm). The filtrate was dried in a vacuum concentrator, re-dissolved in 100 μL of methanol, and used for analysis of metabolite extracts.

Samples were analyzed on a Triple-TOF 5600 System (AB Sciex, Framingham, MA, USA) fitted with a DuoSpray ion source (AB Sciex), which was connected to an ACQUITY UPLC System (Waters, Milford, MA, USA). An aliquot (5 μL) was injected into an RP-C18 column (250 mm × 4.6 mm, Waters) with a column temperature of 30 °C, a wavelength of 318 nm, and a flow rate of 1 mL/min. The mobile phase was 0.1 % acetic acid in water (A) and acetonitrile (B). The gradient was as follows: 0.01 min, 92.0 % A (8.0 % B); 10.00 min, 86.4 % A (13.6 % B); 20.00 min, 84.8 % A (15.2 % B); 30.00 min, 80.8 % A (19.2 % B); 60.00 min, 52.8 % A (47.2 % B); 80.00 min, 5.0 % A (95.0 % B); 90.00 min, 5.0 % A (95.0 % B); 91.00 min, 92.0 % A (8.0 % B); 101.00 min, 92.0 % A (8.0 % B). The column effluent was directed to the electrospray ionization source. The source voltage and source temperature were set to 5500 V and 600 °C for positive ionization and/or 4500 V and 550 °C for negative ionization. The de-clustering potential was 100 V and the collision energy was 35 ± 10 eV. The curtain gas flow was set to 30 arbitrary units and the ion source gas (GS1 and GS2) at 50 psi. The mass range was 100–1000 *m/z* for positive scan mode and 50–1000 *m/z* for negative scan mode. The accumulation time was 0.15 s for MS and 0.08 s for MS/MS. Acquisition of the MS/MS spectra was controlled by the information dependent acquisition function of the Analyst TF software (version 1.5.1; AB Sciex) with the following parameters: dynamic background subtraction, charge monitoring to exclude multiply charged ions/isotopes, and dynamic exclusion of former target ions for 5 s. Mass accuracy was maintained by an automated calibrant delivery system (AB Sciex) interfaced to the second inlet of the DuoSpray source.

Metabolites were identified using PeakView software (version 1.1; AB Sciex) with the ChemSpider Service including PubChem (https://pubchem.ncbi.nlm.nih.gov/), NIST (http://www.nist.gov/), and MassBank (http://www.massbank.jp/). Peaks with a low mass error (<5 ppm) were identified and isotope pattern matching was used to match against the library. MS/MS fragments and isotopic distribution were calculated using PeakView software and matched with entries in the Metlin (http://metlin.scripps.edu/xcms/) and Massbank databases.

### Metabolite extraction and analysis by gas chromatograph time-of-flight mass spectrometry

Each freeze-dried sample (40 mg) was added to 0.5 mL of 75 % methanol (V methanol: V water = 3: 1) containing 20 μL of adonitol (0.2 mg/mL stock in dH_2_O, Sigma, St. Louis, MO, USA) as an internal standard. The resulting mixture was vortexed for 10 s and then homogenized in a ball mill for 3 min at 65 Hz. After centrifuging the samples at 12,000 × g for 15 min at 4 °C, metabolites were extracted from the resulting supernatants. The quality control was prepared by mixing together equal amounts of all the extracts. The extracts were then dried using a vacuum concentrator, and 80 μL of methoxylamine hydrochloride was added. The samples were mixed gently and incubated for 20 min at 80 °C. Subsequently, 100 μL of bis(trimethylsilyl)trifluoroacetamide containing 1 % trimethylchlorosilane were added to each sample, and the samples were incubated for 1 h at 70 °C. The samples were cooled to room temperature and 10 μL of standard fatty acid methyl esters (C8-C16 (1 mg/ml) and C18-C24 (0.5 mg/ml) were mixed in chloroform) were added. The samples were then analyzed by gas chromatograph time-of-flight mass spectrometry (GC-TOF-MS).

GC-TOF-MS analysis was performed on a 7890 GC system (Agilent Technologies, Palo Alto, CA, USA) coupled with a Pegasus HT TOF-MS (LECO, St Joseph, MI, USA) using a Rxi-5Sil MS column (250 μm ID × 30 m; Restek, Bellefonte, PA, USA). Each sample (1 μL) was injected into a splitless injector set at 280 °C and split mode. Helium was used as the carrier gas. The front inlet purge flow was 3 mL min^−1^ and the gas flow rate through the column was 20 mL min^−1^. After incubating at 50 °C for 1 min, the temperature was raised to 330 °C at a rate of 10 °C min^-1^ and then maintained at the final temperature for 5 min. The ion source was set to 220 °C. The energy was -70 eV in electron impact mode. After 366 s of solvent delay, the detector was used in scan mode 50–800 *m/z*. All samples were measured in a randomized manner. Raw peaks were identified using ChromaTOF software (version 4.3X; LECO) from the LECO-Fiehn Rtx5 database (version Rtx5; LECO). The retention time index method was used for peak identification with a retention time tolerance of 5000.

### Protein extraction and analysis by two-dimensional chain electrophoresis (2-DE)

Each freeze-dried sample (2 g) was added to 20 mL of an acetone solution containing 10 % trichloroacetic acid and 10 mM dithiothreitol (DTT). The mixture was incubated for 12 h at -20 °C, and then centrifuged at 20,000 × g for 30 min at 4 °C. The resulting pellet was washed with ice-cold acetone containing 0.07 % DTT, dried using a vacuum concentrator, resuspended in a lysis buffer (9 M urea, 2 M thiourea, 4 % 3-[(3-cholamidopropyl) dimethylammonio]-1-propanesulfonate (CHAPS), 1 % DTT, and 0.5 % immobilized pH gradient (IPG) buffer, pH = 4-7), and sonicated three times for 30 min at 25 °C. The resulting suspension was centrifuged at 20,000 × g for 30 min at 4 °C, and the supernatant was collected as crude extract. Protein concentrations were determined using the Bradford assay [26] with bovine serum albumin as the standard.

Protein (1.6 mg) dissolved in 460 μL rehydration buffer (9 M urea, 4 % CHAPS, 2 M thiourea, 1 % DTT, 0.5 % pH 4-7 IPG buffer and 0.002 % bromophenol blue) was loaded onto pH 4-7 IPG strips (24 cm) and then rehydrated for at least 18 h. Isoelectric focusing (IEF) was completed using the Ettan IPGphor III system (GE Healthcare, Chalfont St. Giles, UK). The IEF ran at 50 V for 1 h (step), 100 V for 2 h (gradual), 150 V for 2 h (gradual), 250 V for 2 h (gradual), 1000 V for 2 h (step), 4000 V for 5 h (gradual), 10,000 V for 6 h (gradual), and then 10,000 V for a total of 110,000 Vh. Following electrofocusing, the protein in the strips was denatured using equilibration buffer (6 M urea, 75 mM Tris-HCl pH 8.8, 29 % glycerol, 2 % sodium dodecyl sulfate, 0.002 % bromophenol blue, and 1.5 % DTT) for 15 min and then incubated for 15 min at room temperature with the equilibration buffer with the DTT replaced with 3.5 % iodoacetamide. The second dimension of 2-DE was performed using 12.5 % polyacrylamide gels and an Ettan DALTsix electrophoresis gel system (GE Healthcare). The proteins were visualized using colloidal Coomassie brilliant blue G-250 using an improved method called “Blue silver” [27].

### Visualization of proteins and image analysis

The Coomassie stained gels obtained as described above were scanned using an ImageScaner III (GE Healthcare) and analyzed using ImageMaster™ 2D Platinum Version 5.0 software (GE Healthcare). Image analysis included subtracting background, and detecting (Saliency = 60, Min area = 15, Smooth = 6), measuring and matching spots. Only spots present on all three replicate gels were further analyzed. All spot volumes were normalized as a percentage of the total volume of all spots on the gel and a Student’s *t*-test was performed to determine the significant differences between two groups. The protein spots were considered significantly increased or decreased when the abundance fold change was more than 1.5 at *P* < 0.05 post-treatment compared with pre-treatment.

### Protein identification and functional analysis

Analysis using matrix-assisted laser desorption ionization time-of-flight mass spectrometry (MALDI-TOF-MS/MS) was performed as previously described with some modifications [28]. Briefly, selected spots were excised from gels, washed twice with ultrapure water and then destained for 30 min at 37 °C using 50 μL of wash solution (50 % acetonitrile and 25 mM ammonium bicarbonate). The gel was dehydrated with 100 μL of 100 % acetonitrile and then incubated in a trypsin solution (1 g/L trypsin in 50 mM acetic acid then diluted with 25 mM ammonium bicarbonate containing 10 % acetonitrile to a concentration of 0.02 μg/μL) in an ice-bath for 30 min, and then covered in 20 μL of 25 mM ammonium bicarbonate containing 10 % acetonitrile. Digestion occurred overnight at 37 °C. The resulting tryptic peptides in the supernatant were extracted using 50 μL of 67 % acetonitrile containing 5 % trifluoroacetic acid at 37 °C for 30 min. All the supernatants were collected, combined and dried in a Speed-Vac after centrifugation.

Tryptic peptides were dissolved in 10 μL of an *R*-cyano-4-hydroxycinnamic acid saturated solution (*R*-cyano-4-hydroxycinnamic acid in 0.1 % trifluoroacetic acid and 50 % acetonitrile) and passed through C18 Zip-Tips. Samples (1 μL) were analyzed by ABI 4800 MALDI-TOF/TOF (Applied Biosystems, Foster City, CA, USA). The UV laser was operated at a 200 Hz repetition rate with a wavelength of 355 nm, and the accelerated voltage at 20 kV. Myoglobin digested by trypsin was used to calibrate the mass instrument with the internal calibration mode. The range of parent mass peaks was 800–3500 Da and a minimum S/N 20 was selected for analysis by tandem TOF/TOF. To examine against available databases, MS and MS/MS spectra were submitted to MASCOT 2.1 (http://www.matrixscience.com) by GPS 3.6 Explorer software (Applied Biosystems). The search parameters were as follows: NCBInr database, taxonomy of green plants, trypsin digested with one missing cleavage, fixed modifications of carbamidomethyl, variable modifications of oxidation, MS tolerance of 0.15 Da, and MS/MS tolerance of 0.25 Da. Known contaminants, e.g. keratin, were excluded. The MASCOT score was based on a combination of MS and MS/MS spectra, where higher than 40 was considered statistically significant (*P* < 0.05).

Protein functions were categorized using MapMan (http://mapman.gabipd.org/) bin codes [29]. The predication of identified proteins derived from *C. terniflora* leaves was performed by transferring annotations to the *Arabidopsis* genome with consideration of orthologous genes. Identified proteins were mapped to pathways using the Kyoto Encyclopedia of Genes and Genomes (KEGG) database (http://www.genome.jp/kegg/) [30].

### Transcript analysis using quantitative reverse transcription polymerase chain reaction (qRT-PCR)

Total RNA was extracted from leaves using a RNA isolation kit (Huayueyang, Beijing, China), and served as the template by which to synthetize cDNA using 5X All-In-One RT MasterMix (with AccuRT Genomic DNA Removal Kit) (Applied Biological Materials, Richmond, BC, Canada) according to the manufacturer’s instructions. qRT-PCR was then performed in a Bio-Rad IQ2 Multicolor Real-Time PCR Detection System (Bio-Rad, Hercules, CA, USA) with EvaGreen 2X qPCR Master Mix-iCycler (Applied Biological Materials) as the fluorescent dye. *GAPDH* served as a housekeeping gene to normalize target gene quantities. The gene specific primers are listed in Additional file 1: Table S1. Three biological replicates were performed, and the relative expression levels were calculated using the 2^-ΔΔCt^ method.

### Enzyme activity assays

#### L-Serine deaminase

L-serine deaminase activity was measured based on a method published by Wood et al. [31] with minor modifications. *C. terniflora* leaves were collected pre- and post-treatment with high level UV-B irradiation for 5 h with or without a subsequent 36 h incubation in the dark. The leaves (500 mg) were frozen and then homogenized on ice using a mortar and pestle in 5 mL of extraction buffer (0.1 M Tris-HCl (pH 7.4), 1 mM ethylenediaminetetraacetic acid disodium salt, and 0.1 mM 2- mercaptoethanol). The resulting homogenate was centrifuged at 13,000 × g for 30 min at 4 °C. Supernatants, 400 μL each, were collected, and mixed with 560 μL reaction buffer (20 mM Tris-HCl (pH 8.5), 20 μM pyridoxal phosphate, and 20 mM L-serine). Before adding the enzyme and substrate, the assay tubes were warmed to 37 °C. After adding the substrate, the reaction was allowed to proceed for 1 h and then was stopped using 0.2 mL of 100 % trichloroacetic acid. The pyruvate in the samples was quantified using a Pyruvate assay kit (Nanjing Jiancheng, Jiangsu, China). Protein concentrations were determined using the Bradford assay [26] with bovine serum albumin as the standard. A unit of L-SD activity was arbitrarily defined as the amount of enzyme necessary to form 1 μM pyruvate of 1 mg protein in 1 h under the above experimental conditions.

#### L-Tryptophan synthase

L-tryptophan synthase activity was measured based on the method published by Last et al. [32] with minor modifications. Briefly, the samples for analysis were collected as described above. The collected leaves (1 g) were frozen and homogenized on ice using a mortar and pestle in 5 mL extraction buffer (0.1 M potassium phosphate (pH 8.2) and 50 mg polyvinylpolypyrrolidone). The homogenate was centrifuged at 13,000 × g for 30 min at 4 °C. Supernatants (400 μL) were collected, and mixed with 570 μL reaction buffer (80 μM potassium phosphate (pH 8.2), 10 μM pyridoxal phosphate, 10 mM L-serine, and 20 μM indole). Before adding the enzyme and substrate, the assay tubes were warmed to 30 °C. After adding the substrate, the reaction was allowed to proceed for 3 h and then stopped with 0.1 mL of 0.2 M sodium hydroxide. The residual indole was extracted into 0.8 mL toluene by vortexing the sample gently. After centrifuging for 15 min at 1500 × g, 0.4 mL of the toluene layer was added to 0.4 mL of ethanol and 1 mL of Kovacs (0.4 g 4-(dimethylamino) benzaldehyde dissolved in 38 mL ethanol and 8 mL hydrochloric acid). The color was allowed to develop for 30 min at room temperature and the product was then measured by spectrophotometer at 540 nm. Protein concentrations were determined using the Bradford assay [26] with bovine serum albumin as the standard. A unit of L- tryptophan synthase activity was arbitrarily defined as the amount of enzyme necessary to consume 1 μM indole of 1 mg protein in 1 h under the above experimental conditions.

### Statistical analysis

The SPSS statistical software (version 22.0; IBM, Armonk, NY, USA) was used for statistical evaluation. One-Way ANOVA followed by Tukey’s multiple comparison post-hoc tests was performed and the Student’s *t*-test was also used when only two groups were compared. All results were shown as mean ± SD from three independent biological replicates. *P* < 0.05 was considered a statistically significant difference.

## Results

### Accumulation of indole alkaloids in *Clematis terniflora* DC. leaves in response to exposure to high level of UV-B irradiation and the dark

**Fig. 1:**
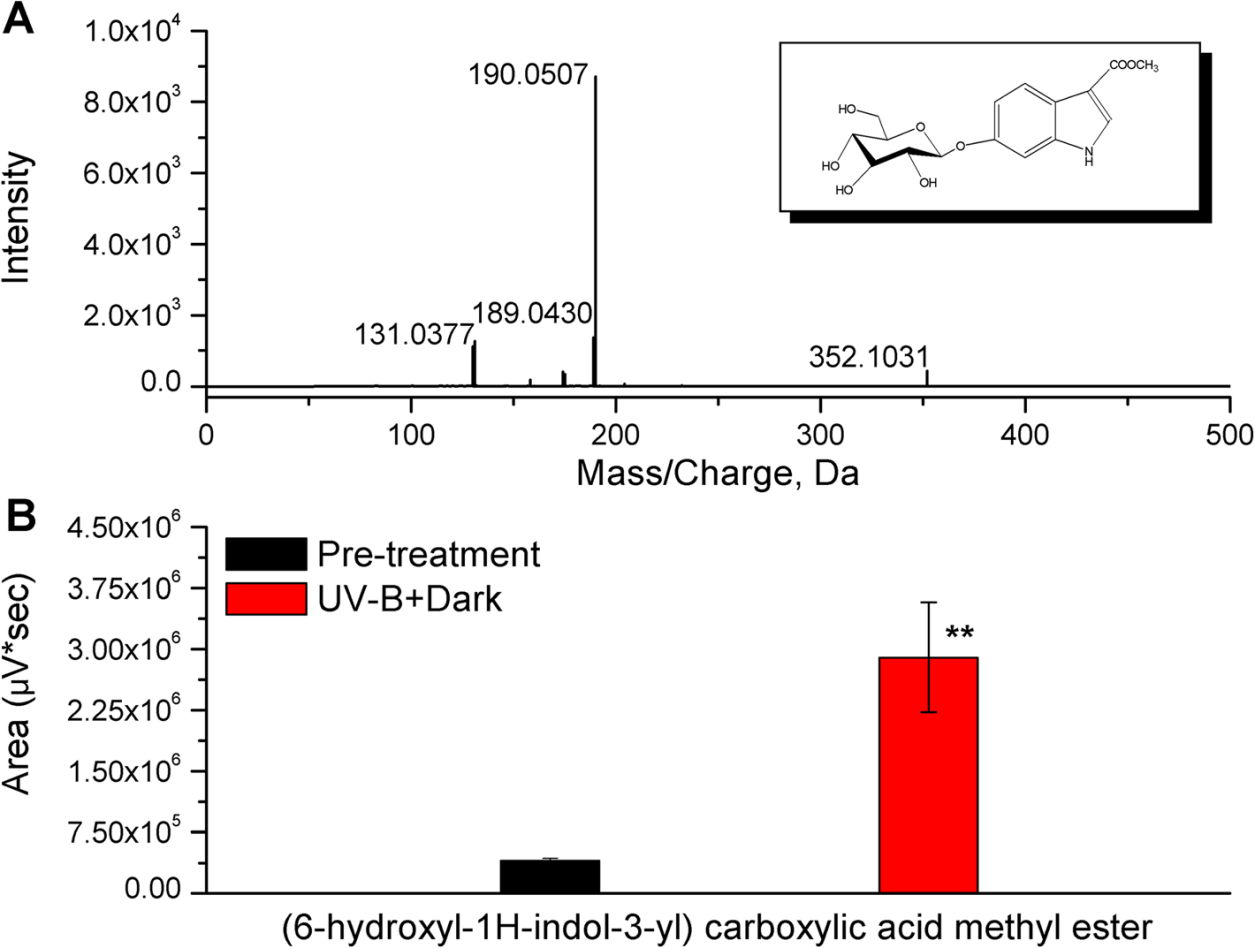
Identification of (6-hydroxyl-1H-indol-3-yl) carboxylic acid methyl ester in *Clematis terniflora* DC. leaves. Methanol extracts were obtained from *C. terniflora* leaves pre- and post-treatment with high level of UV-B irradiation for 5 h followed by an incubation for 36 h in the dark (UV-B + Dark), and then analyzed using HPLC-TOF-MS/MS. **a** HPLC-TOF-MS/MS spectrum of (6-hydroxyl-1H-indol-3-yl) carboxylic acid methyl ester (negative mode) post-treatment. **b** Statistics of peak area of (6-hydroxyl-1H-indol-3-yl) carboxylic acid methyl ester pre- and post-treatment. Data are shown as the mean ± SD from three independent biological replicates. Asterisks indicate significant differences as measured by Student’s *t*-test (**P < 0.01)

To investigate the effect of high level of UV-B irradiation and the dark on the amount of plant indole alkaloids, the metabolite (6-hydroxyl-1H-indol-3-yl) carboxylic acid methyl ester was identified and quantified using HPLC-TOF-MS/MS. *C. terniflora* leaves were collected pre- and post-exposure to UV-B irradiation and the dark. (6-hydroxyl-1H-indol-3-yl) carboxylic acid methyl ester was discovered in the metabolomic profile and the peak area was evaluated. The spectrum of the indole alkaloid of negative-ion showed the quasi-molecular ion at *m/z* 352.1031 ([M-H]^-^) and a fragment at *m/z* 190.0507 ([M-H-162]^-^) (Fig. 1a). The peak area of (6-hydroxyl-1H-indol-3-yl) carboxylic acid methyl ester increased 7-fold in *C. terniflora* leaves post-treatment compared with pre-treatment (Fig. 1b). Therefore, the indole alkaloid biosynthesis pathway may be activated by high level of UV-B irradiation followed by the dark in *C. terniflora* leaves.

### Perturbation of metabolic processes based on protein level in *Clematis terniflora* DC. leaves after exposure to high level of UV-B irradiation and the dark

**Fig. 2:**
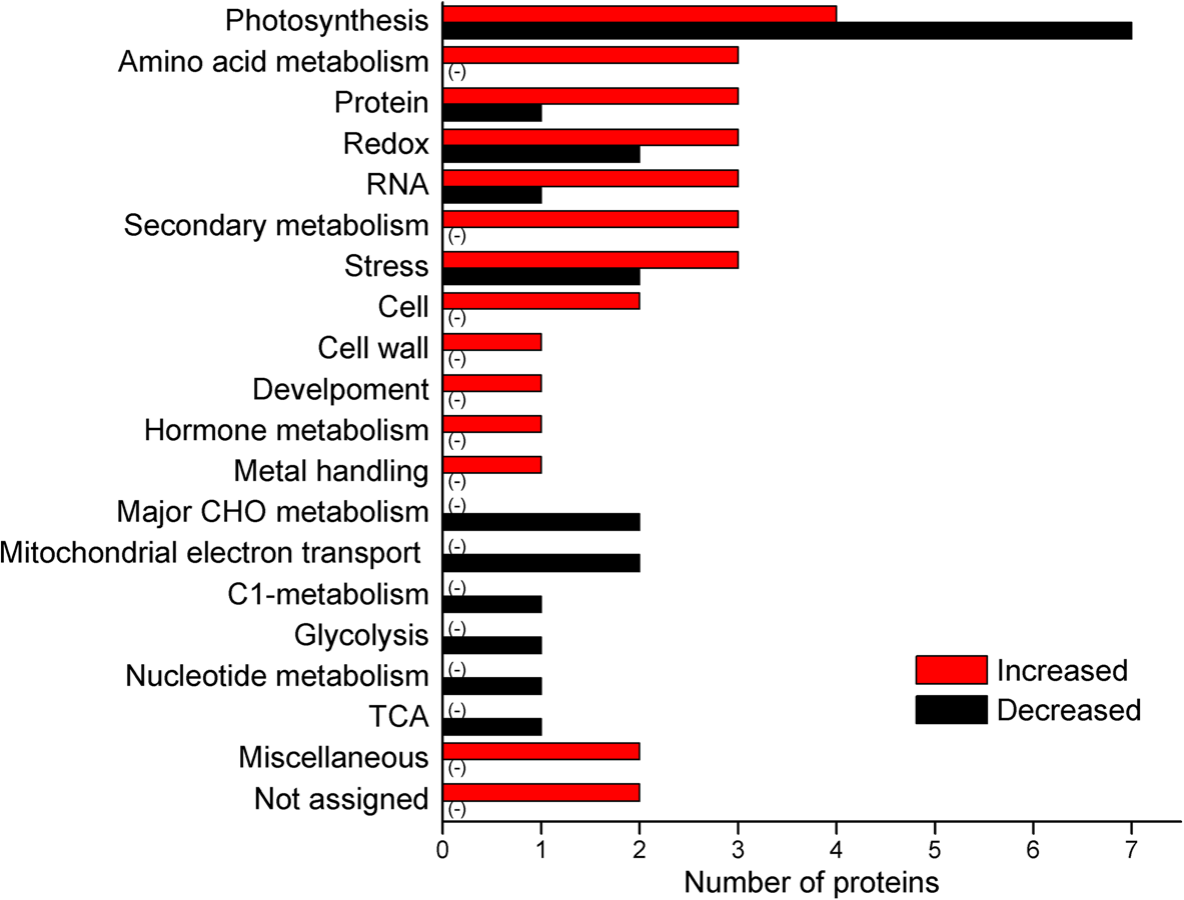
Functional distribution of proteins identified in *Clematis terniflora* DC. leaves. *C. terniflora* leaves were collected pre- and post-treatment with high level of UV-B irradiation for 5 h followed by dark for 36 h. Proteins within the leaves were identified using a 2-DE gel proteomic technique based on MALDI-TOF-MS/MS. The function of each identified protein was predicated and categorized using MapMan bin codes. Numbers of categorized proteins are shown in the graph. Red bars indicate increased proteins post-treatment compared with pre-treatment, and black bars indicate decreased. Abbreviations: CHO, carbohydrate; TCA, tricarboxylic acid

To investigate the perturbation of metabolic processes in leaves in response to high level of UV-B irradiation and an incubation in the dark, proteomic analysis was performed using 2-DE technique. *C. terniflora* leaves were collected pre- and post-treatment. Proteins spots with changes of at least 1.5-fold compared with a control gel were selected for identification by MALDI-TOF-MS/MS. Data were analyzed using the NCBInr database. A total of 53 proteins were identified, including 32 that increased and 21 that decreased post-treatment compared with pre-treatment (Additional file 2: Table S2). Identified proteins were predicated by transferring annotations to the *Arabidopsis* genome and functional categories were assigned using MapMan bin codes. Based on the functional analysis, proteins related to amino acid metabolism, protein metabolism, RNA, secondary metabolism, cell, cell wall, development, hormone metabolism, and metal handing increased in *C. terniflora* leaves in response to high level of UV-B irradiation and the dark (Fig. 2). Meanwhile, proteins related to photosynthesis, major carbohydrate (CHO) metabolism, and mitochondrial electron transport decreased in leaves of *C. terniflora* in response to high level of UV-B irradiation and the dark (Fig. 2). Notably, the decreased number of proteins related to photosynthesis was more than 1.7-fold compared with increased number, while, in particular, the proteins related to amino acid metabolism all increased after exposure to high level of UV-B irradiation and the dark compared with pre-treatment.

### Perturbation of metabolic processes based on metabolite level in *Clematis terniflora* DC. leaves after exposure to high level of UV-B irradiation and the dark

**Fig. 3:**
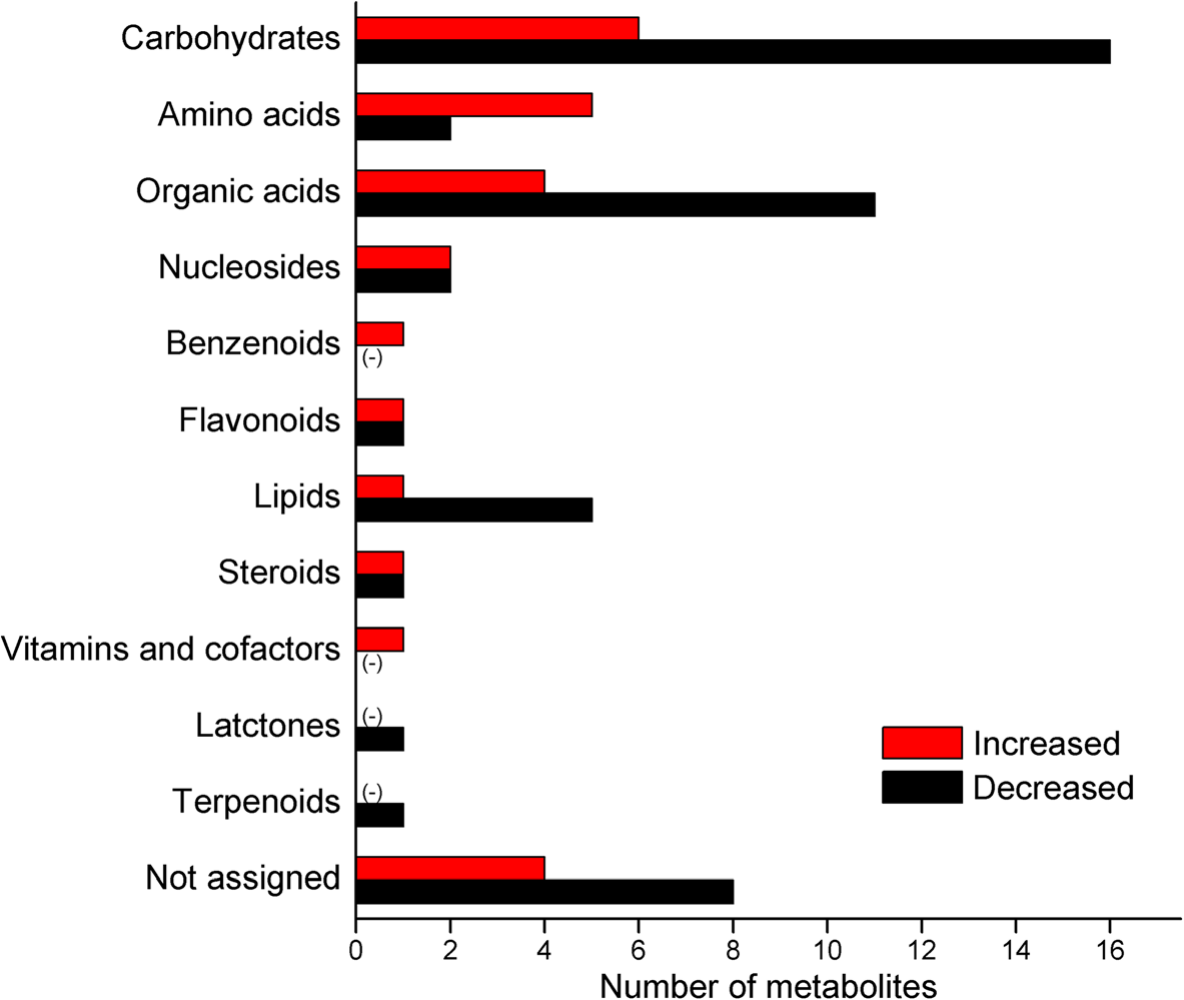
Metabolic distribution of metabolites identified in *Clematis terniflora* DC. leaves. *C. terniflora* leaves were collected pre- and post-treatment with high level of UV-B irradiation for 5 h followed by the dark for 36 h. Metabolites were extracted using 75 % methanol and analyzed using GC-TOF-MS. Metabolites were functionally categorized using the KEGG database or manually classified based on their chemical structures. Numbers of categorized metabolites are shown in the graph. Red bars indicate increased metabolites post-treatment compared with pre-treatment, and black bars indicate decreased

A GC-TOF-MS-based metabolomic technique was employed to reveal any perturbations in metabolites due to exposure to high level of UV-B irradiation followed by the dark. *C. terniflora* leaves were collected pre- and post-treatment. In total, 139 metabolites were identified, of which, 74 changed in response to the binary stress. Of the affected metabolites, 26 accumulated and 48 decreased (Additional file 3: Table S3). Based on KEGG annotation and manual identification, the affected metabolites were classified into 12 categories (Fig. 3). Metabolites that experienced an increase following treatment were more likely to be involved in amino acids, benzenoids, vitamins and cofactors than those that decreased, while vice versa was true for metabolites involved in carbohydrates, organic acids, and lipids (Fig. 3). Specifically, the results demonstrate that amino acid metabolism was enhanced and carbohydrate metabolism was inhibited in response to high level of UV-B irradiation followed by the dark.

### Activation of amino acid metabolism

**Fig. 4:**
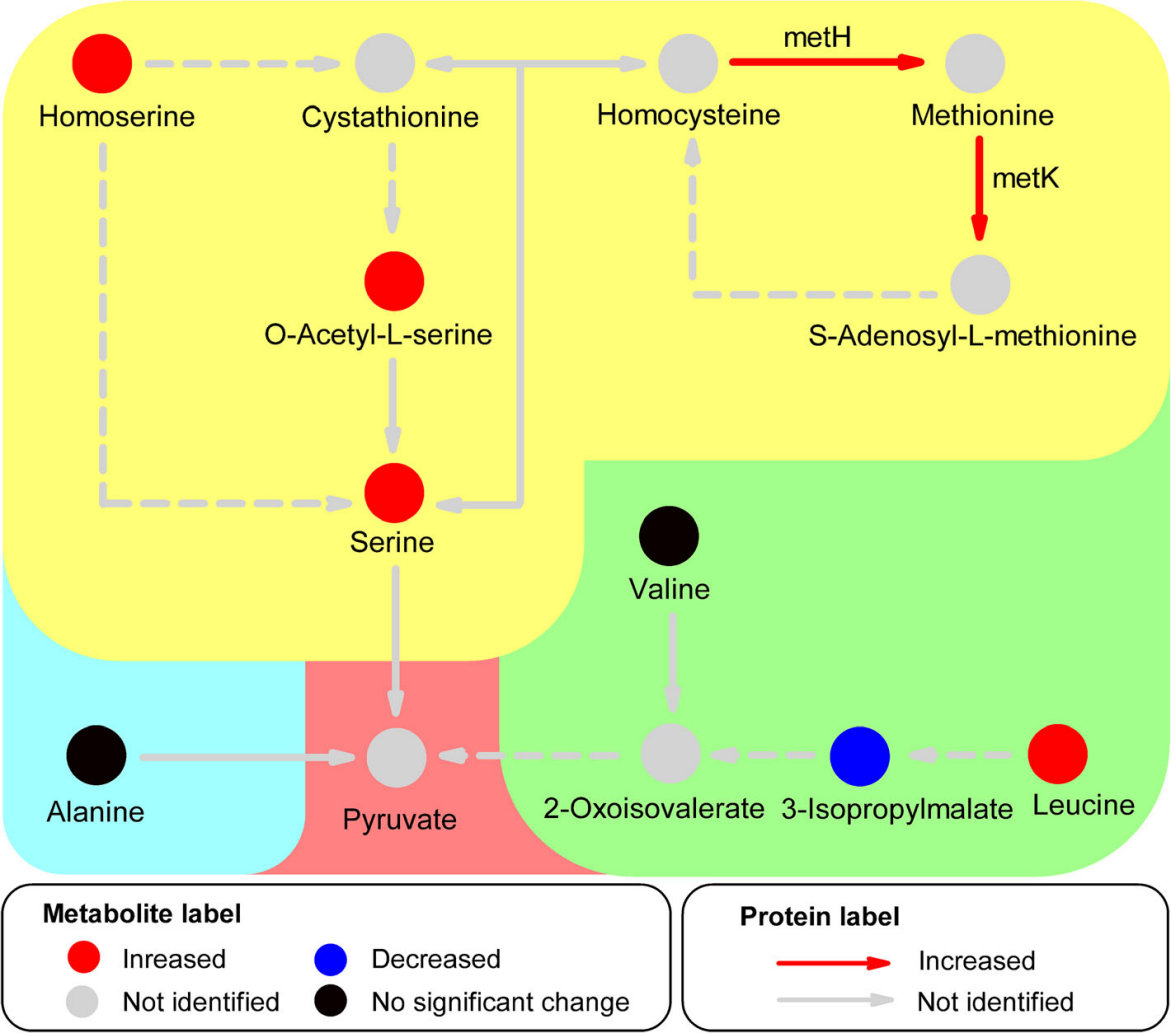
Simplified amino acid metabolism pathway based on KEGG analysis of metabolites and proteins identified in *C. terniflora* leaves pre- and post-treatment with high level of UV-B irradiation for 5 h followed by the dark for 36 h. Circles and arrows indicate metabolites and proteins, and red and blue represent significant increases and decreases, respectively, in metabolites and proteins. Black indicates no significant change and gray indicates those that were unidentified. Abbreviations: metH, 5-Methyltetrahydropteroyltriglutamate-homocysteine methyltransferase; metK, S-adenosyl-L-methionine synthetase

Based on proteomic and metabolomic analyses, many of the identified proteins and metabolites related to amino acid metabolism increased in response to high level of UV-B irradiation followed by the dark. To summarize perturbations in response to this stress, proteins and metabolites were mapped onto the amino acid metabolism pathway using the KEGG database (Fig. 4). 5-Methyltetrahydropteroyltriglutamate-homocysteine methyltransferase (metH), which transforms homocysteine into methionine, experienced a more than 1.7-fold increase post-treatment compared with pre-treatment. S-adenosyl-L-methionine synthetase (metK) increased 1.6-fold after exposure to high level of UV-B irradiation followed by the dark. Six amino acids were identified in this pathway and 4 of these amino acids increased more than 1.5-fold. O-acetyl-L-serine and serine, which are in the same metabolic pathway, increased 18- and 3-fold post-treatment with exposure to high level of UV-B irradiation followed by the dark compared with pre-treatment, respectively, while, homoserine and leucine increased 7- and 127-fold, respectively. Meanwhile, valine and alanine remained unchanged after exposure to high level of UV-B irradiation and the dark.

### Enhancement of transcriptional level in indole biosynthesis (shikimate metabolic) pathway

**Fig. 5:**
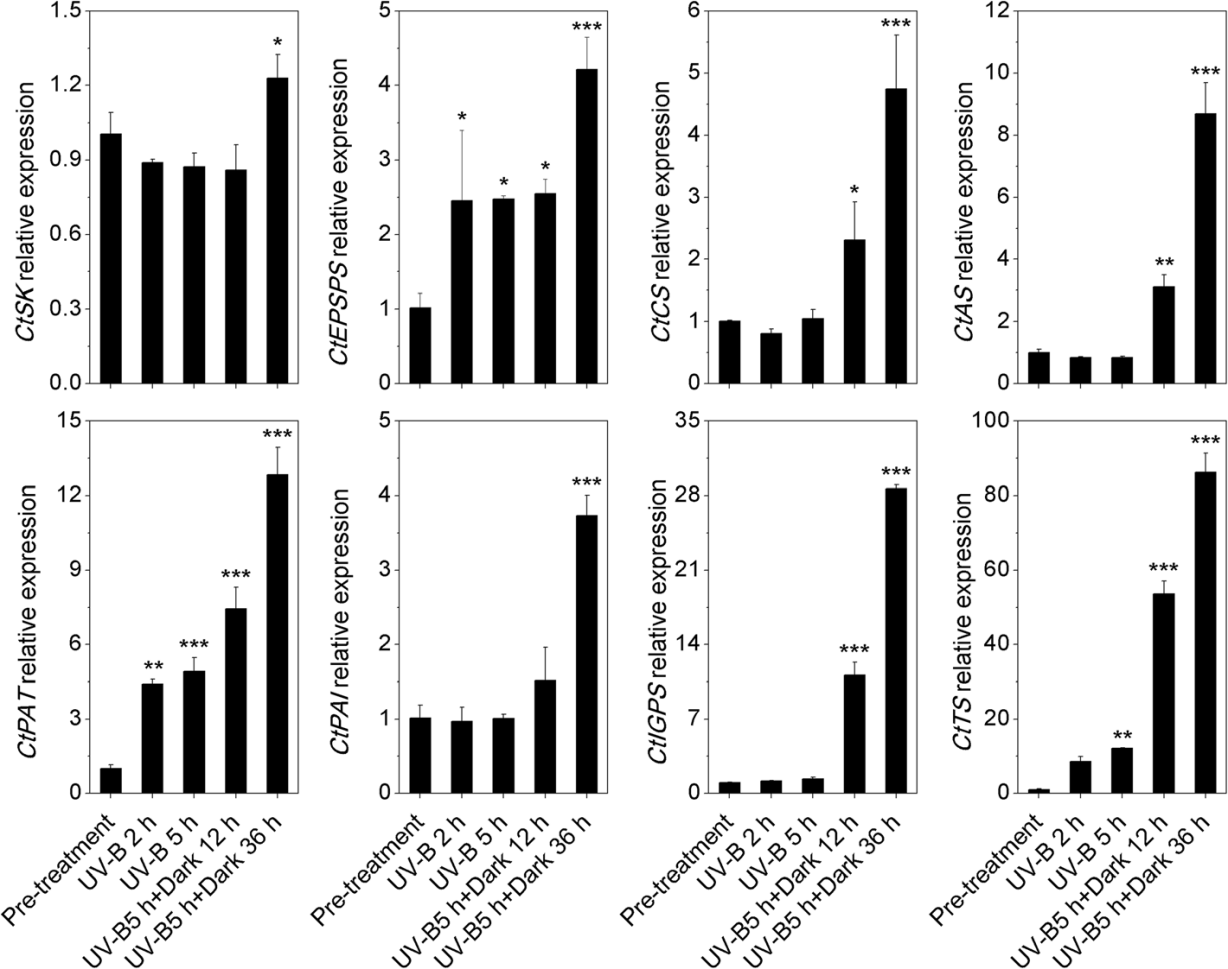
Analysis of transcripts involved in the shikimate metabolic and indole/tryptophan pathways in *Clematis terniflora* DC. leaves by qRT-PCR pre- and post-treatment with high level of UV-B irradiation for 2 h (UV-B 2 h), 5 h (UV-B 5 h), 5 h followed by an incubation for 12 h in the dark (UV-B 5 h + Dark 12 h), and 5 h followed by an incubation for 36 h in the dark (UV-B 5 h + Dark 36 h). Abbreviations: *SK*, shikimate kinase; *EPSPS*, 5-enolpyruvylshikimate-3-phosphate synthase; *CS*, chorismate synthase; *AS*, anthranilate synthase; *PAT*, phosphoribosylanthranilate transferase; *PAI*, phosphoribosylanthranilate isomerase; *IGPS*, indole-3-glycerol phosphate synthase; *TS*, tryptophan synthase. Data are shown as the mean ± SD from three independent biological replicates. Asterisks indicate significant changes as measured by One-Way ANOVA test (*P < 0.05; **P < 0.01; ***P < 0.001)

To investigate the response of the indole biosynthesis (shikimate metabolic) pathway to high level of UV-B irradiation and the dark, qRT-PCR-based transcriptomic analysis was performed. *C. terniflora* leaves were collected pre- and post-treatment with high level of UV-B irradiation for 2 or 5 h, or high level of UV-B irradiation for 5 h followed by an incubation in the dark for 12 or 36 h. The relative expression of 8 genes involved in the metabolic processes going from shikimate to L-tryptophan was upregulated (Fig. 5). One group of genes involved in shikimate metabolic pathway were as follows: shikimate kinase (*CtSK*) was upregulated after high level of UV-B irradiation for 5 with 36 h in the dark; 5-enolpyruvylshikimate-3-phosphate synthase (*CtEPSPS*) was upregulated post-treatment; chorismate synthase (*CtCS*) was upregulated after high level of UV-B irradiation for 5 h either 12 or 36 h in the dark compared with pre-treatment. Other groups of genes involved in the biosynthesis of indole nuclei were anthranilate synthase (*CtAS*), which was upregulated after 5 h UV-B and dark for 12 or 36 h; phosphoribosylanthranilate transferase (*CtPAT*), which was upregulated at all points; phosphoribosylanthranilate isomerase (*CtPAI*), which was upregulated after 5 h UV-B and the dark for 36 h; and indole-3-glycerol phosphate synthase (*CtIGPS*), which was upregulated after 5 h UV-B and 12 or 36 h in the dark. Additionally, the relative expression of tryptophan synthase (*CtTS*), which is involved in the formation of indole and L-tryptophan, was upregulated 12-, 53- and 86-fold after 5 h UV-B, 5 h UV-B with 12 h in the dark, and 5 h UV-B with 36 h in the dark compared with pre-treatment, respectively.

### Accumulation of metabolites in the indole biosynthesis pathway

**Fig. 6:**
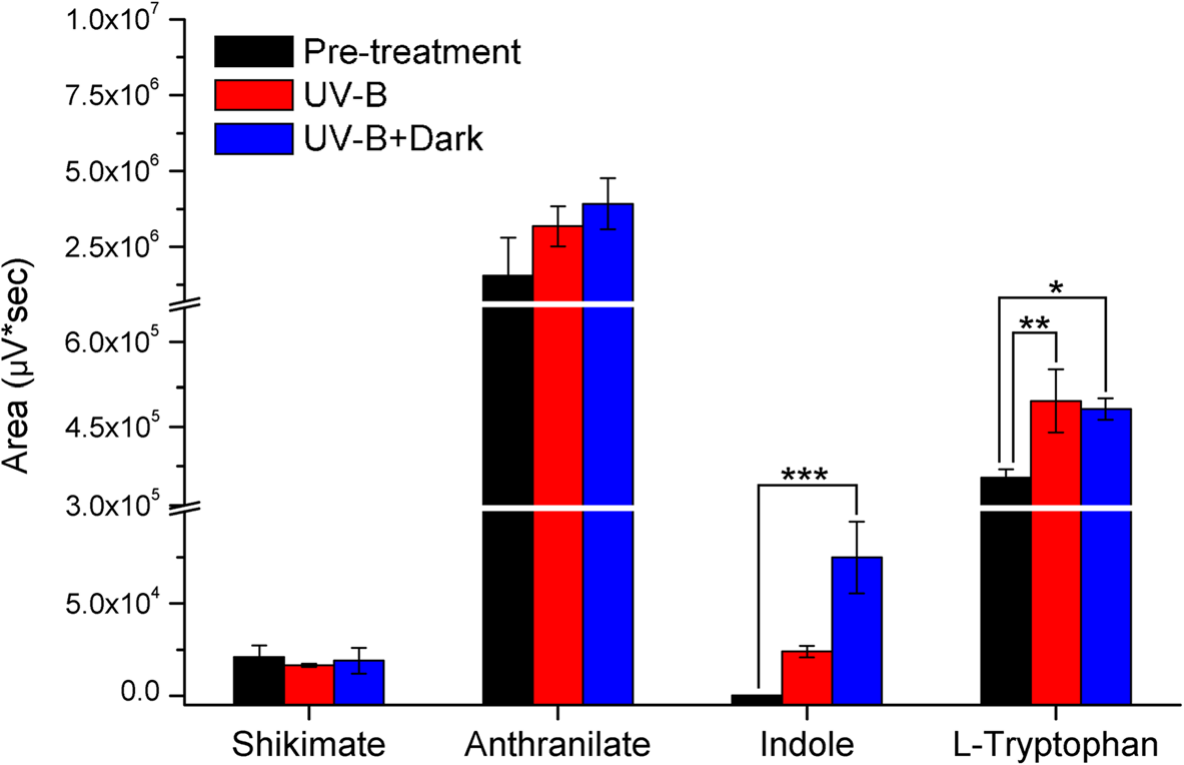
Statistics of area of shikimate, anthranilate, indole, and L-tryptophan HPLC-TOF-MS/MS peaks in *Clematis terniflora* DC. leaves pre- and post-treatment with high level of UV-B irradiation for 5 h with or without an incubation for 36 h in the dark. Data are shown as the mean ± SD from three independent biological replicates. Asterisks indicate significant changes measured by One-Way ANOVA test (*P < 0.05; **P < 0.01; ***P < 0.001)

To verify the results of the proteomic, metabolomic and transcriptomic analyses, metabolites involved in the indole biosynthesis pathway were determined using HPLC-TOF-MS/MS technique. *C. terniflora* leaves were collected pre- and post-treatment with high level of UV-B irradiation for 5 h with or without a subsequent incubation in the dark for 36 h. The 4 metabolites of shikimate, anthranilate, indole, and L-tryptophan were selected for further analysis (Fig. 6). Shikimate showed no significant changes after 5 h UV-B, or 5 h UV-B with 36 h dark compared with pre-treatment. Although anthranilate did not respond to high level of UV-B irradiation with the dark, it remained stable at a high concentration under this binary stress. Indole increased 141- and 441-fold after 5 h UV-B, and 5 h UV-B with 36 h in the dark compared with pre-treatment, respectively. L-tryptophan increased 1-fold both after 5 h UV-B and 5 h UV-B with 36 h in the dark compared with pre-treatment.

### Increase in the activity of enzymes involved in indole biosynthesis

**Fig. 7:**
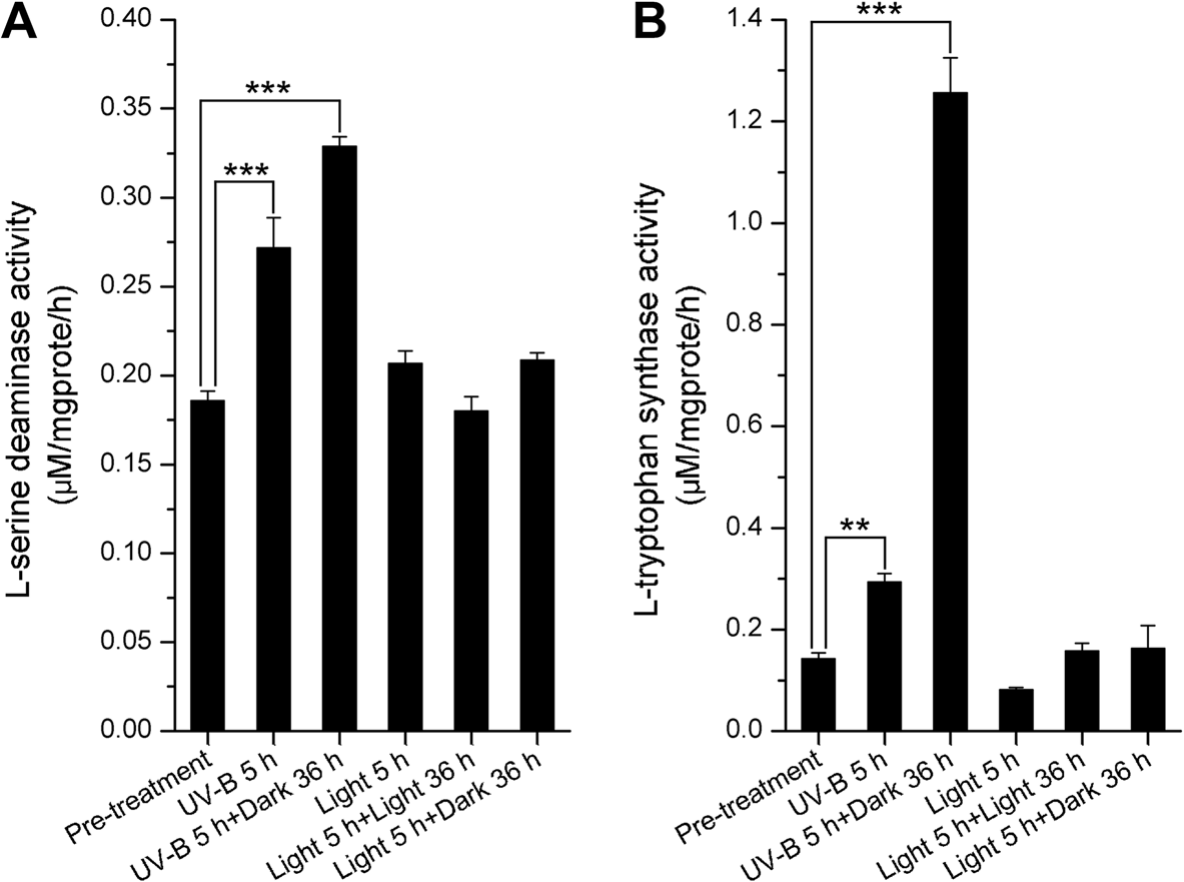
Enzymatic activity of (**a**) L-serine deaminase and (**b**) L-tryptophan synthase in *Clematis terniflora* DC. leaves pre- and post-treatment with high level of UV-B irradiation for 5 h (UV-B 5 h), high level of UV-B irradiation 5 h followed by an incubation for 36 h in the dark (UV-B 5 h + Dark 36 h), light irradiation for 5 h (Light 5 h), light irradiation for 5 h followed by light irradiation for 36 h (Light 5 h + Light 36 h), or light irradiation for 5 h followed by an incubation in the dark for 36 h (Light 5 h + Dark 36 h). Data are shown as the mean ± SD from three independent biological replicates. Asterisks indicate significant changes measured by One-Way ANOVA test (**P < 0.01; ***P < 0.001)

The activity of L-SD, which transforms serine into pyruvate, was measured to better understand the change in the shikimate metabolic pathway caused by the increase in amino acid metabolism. The results displayed in Fig. 7a show that L-SD activity increased significantly after 5 h UV-B and 5 h UV-B followed by 36 h in the dark compared with pre-treatment. Meanwhile, treatment with light irradiation for 5 h, light irradiation for 5 h followed by light irradiation for 36 h, and light irradiation for 5 h followed by 36 h in the dark had no effect on L-SD activity.

L-tryptophan synthase activity was measured to study the biosynthesis of indole and L-tryptophan, both of which are derived from the shikimate metabolic pathway. L-tryptophan synthase activity increased notably after 5 h UV-B and 5 h UV-B followed by 36 h in the dark (Fig. 7b). However, there were no differences after treatment with light irradiation for 5 h, light irradiation for 5 h followed by light irradiation for 36 h, and light irradiation for 5 h followed by the dark for 36 h compared with pre-treatment (Fig. 7b).

## Discussion

### Enhanced flow from amino acid metabolism pathway into shikimate metabolic pathway

Amino acid metabolism is of considerable interest due the critical roles it plays in cell structure and physiology. Amino acids accumulate in plant species in response to environmental stresses and have diverse biological functions, such as in redox balance [33], anti-oxidant defence [34], and metal binding [35]. In addition, amino acids serve as precursors for a multitude of natural products, including pigments, alkaloids, hormones, and cell well components [36]. Previously published studies have investigated the activation of amino acid metabolism in *C. terniflora* after high level of UV-B irradiation and followed by an incubation in the dark, and it was found amino acid metabolism increased the anti-oxidative capabilities of leaves in response to the binary stress [20]. In this study, an increase in certain proteins and metabolites, e.g. metH and metK, indicates the activation of amino acid metabolism in *C. terniflora* leaves under the binary stress (Figs. 2, 3, and 4). For example, metH catalyzes the formation of methionine and metK catalyzes the synthesis of S-adenosyl-L-methionine, the latter of which is an important primary methyl group donor in biological systems [37]. These results indicate that transmethylation is activated in response to the binary stress and promotes amino acid metabolism.

Interestingly, it was found photosynthesis decreased in *C. terniflora* leaves after treatment with UV-B irradiation and the dark (Fig. 2). Previous work has shown that the inhibition of photosynthesis is indicative of an increase of photorespiration in barley under drought stress [38]. Photorespiration provides metabolites, such as pyruvate, for important metabolic processes involved in protection against stress [39, 40]. L-SD catalyzes the degradation of serine into pyruvate [41] and its activity increased considerably in the presence of leucine [42]. Studies on wheat and *Arabidopsis* have demonstrated an accumulation of leucine following UV-B irradiation [43, 44]. In this current study, serine and L-SD activity increased by high level of UV-B irradiation followed by an incubation in the dark (Figs. 4 and 7a). This indicates that pyruvate synthesis might occur at the expense of serine through the activity of L-SD in *C. terniflora* leaves in response to the experimental stress in this study. The promotion of pyruvate metabolism prevents a shortage in the availability of carbon for photorespiration under the binary stress.

### Indole/tryptophan accumulated through enhancement of shikimate metabolic pathway in response to exposure to high level of UV-B irradiation and the dark

Pyruvate accumulates as a carbon supply for the C3 skeleton needed for many physiological processes in plant cells under stress [45]. It can be metabolized into phosphoenolpyruvate, which enhances shikimate metabolic process [46]. The shikimate metabolic pathway is a well-characterized metabolic sequence that connects central carbon metabolism and downstream pathways by converting phosphoenolpyruvate and erythrose 4-phosphate into chorismate, which is the universal precursor for all aromatic amino acids (AAAs) and numerous secondary metabolites [36, 47]. EPSPS and CS are enzymes that participate in enzymatic steps of the shikimate metabolic pathway and create 5-O-(1-carboxyvinyl)-3-phosphoshikimate and chorismate, respectively. It has been demonstrated that *EPSPS* is upregulated in a high stress-tolerant variety of *Allium cepa* [48]. The induction of shikimate metabolic pathway through the upregulation of *EPSPS* and *CS* in elicitor-treated tomato cells caters to the demand for downstream secondary metabolites [49]. In this study, *EPSPS* was upregulated by high level of UV-B irradiation followed by exposure to the dark (Fig. 5), and *CS* was induced by the dark (Fig. 5), indicating that shikimate metabolic pathway is enhanced in response to this stress.

**Fig. 8:**
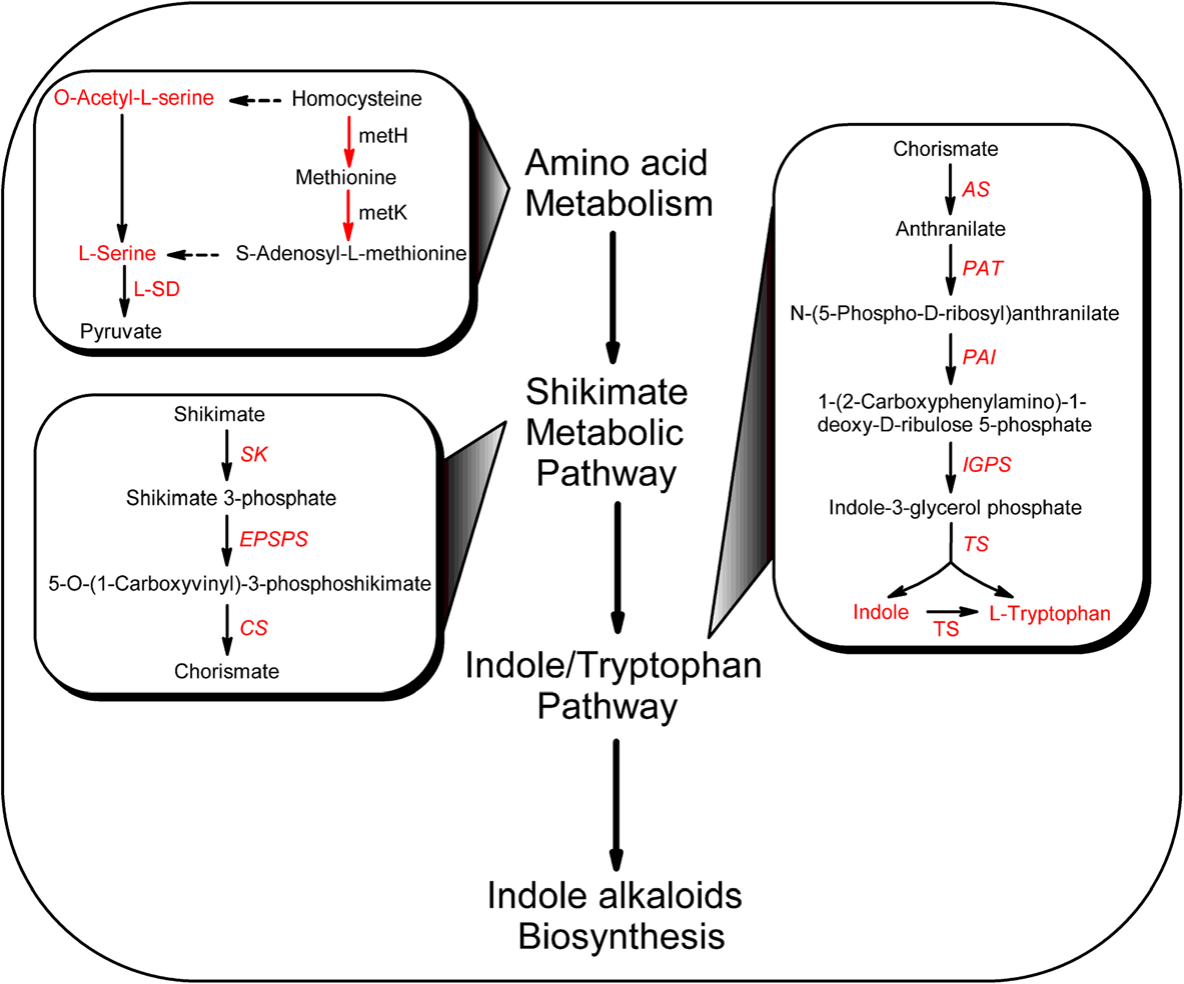
Overview of the metabolic process from amino acid metabolism to shikimate metabolic pathway and downstream indole/tryptophan pathway in *Clematis terniflora* DC. leaves exposed to high level of UV-B irradiation and the dark. Red color indicates an increase. Abbreviations: metH, 5-Methyltetrahydropteroyltriglutamate-homocysteine methyltransferase; metK, S-adenosyl-L-methionine synthetase; L-SD, L-serine deaminase; SK, shikimate kinase; EPSPS, 5-enolpyruvylshikimate-3-phosphate synthase; CS, chorismate synthase; AS, anthranilate synthase; PAT, phosphoribosylanthranilate transferase; PAI, phosphoribosylanthranilate isomerase; IGPS, indole-3-glycerol phosphate synthase; TS, tryptophan synthase

The metabolic pathways downstream of shikimate primarily synthesize L-tryptophan (Trp), L-phenylalanine (Phe), and L-tyrosine (Tyr). Selective modulation of the biosynthesis of these AAAs depends on key branch point enzymes, including chorismate mutase (CM) for Phe and Tyr biosynthesis, and AS for Trp biosynthesis [36]. It has been published that *CM* is induced under the influence of methyl jasmonate in *Vitis vinifera* cells [50], and *AS* is upregulated by cold stress in rice [51]. Interestingly, the Trp, Phe, and Tyr branches of the AAA biosynthesis pathway respond differently to UV-B stress depending on the state of plant growth and degree of damage [52]. Studies on *Arabidopsis* have shown that all of these branches increase in response to UV-B irradiation, and tryptophan in particular accumulates under this stress [43], likely due to the upregulation of *AS* [53]. IGPS is an important enzyme in the biosynthesis of Trp and the hormone indole-3-acetic acid (IAA), in particular, because it is the only known enzyme that catalyzes the formation of the indole ring [54]. The expression of *AS*, *PAT*, *PAI*, *IGPS*, and *TS* suggests concurrently upregulation in response to high level of UV-B irradiation followed by exposure to the dark (Fig. 5). Moreover, the activity of TS was notably elevated post-treatment compared with pre-treatment (Fig. 7b), suggesting activation of the Trp specific pathway through the shikimate metabolic pathway (Fig. 8).

As described above, shikimate is an indispensable metabolite that supplements several metabolic processes. The enhancement of shikimate metabolic pathway on transcriptional level guarantees the stable replenishment of shikimate for different metabolic pathways in a complicated manner. Studies on *Arabidopsis* have shown that the Trp, Phe, and Tyr pathways downstream of shikimate metabolic pathway became elevated at the same time following UV-B irradiation, while the amount of shikimate remained unchanged [43]. In addition, phenylalanine ammonia-lyase (PAL) increased in *C. terniflora* post-treatment compared with pre-treatment (Additional file 2: Table S2), indicating the enhancement of phenylpropanoid metabolic process [55]. Anthranilate not only serves in the formation of tryptophan, but also supplies the biosynthesis of acridone alkaloid [56]. The high level of anthranilate and the stable accumulation of shikimate and anthranilate in *C. terniflora* leaves after exposure to high level of UV-B irradiation followed by the dark (Fig. 6) likely aided in the metabolic process from shikimate to tryptophan.

### Predicted biosynthesis of indole alkaloids based on the elevation of indole/tryptophan after high level of UV-B irradiation and the dark

Current data indicates that high level of UV-B irradiation followed by the dark promote the production of shikimate and the shikimate metabolic pathway for the downstream biosynthesis of tryptophan. During this process, indole is considered a pivotal component in plant physiology when dealing with ongoing stresses [57]. Additionally, it has been suggested that indoles have anti-oxidant and radical scavenging activities [58]. Tryptophan, produced from indole through L-tryptophan synthase, has important biological roles in stress-defense, such as against salinity [59] and water stresses [60]. Indole and tryptophan provide the skeletons for indole alkaloids [61] and IAA [62, 63]. IAA is characterized as a naturally occurring auxin. Notably, IAA is essential not only for plant growth [64], but also regulating plant responses to stresses, such as drought tolerance [65]. Indole alkaloids, secondary metabolite with a role in defense against stress resistance [66, 67], are synthesized from indole and tryptophan [61]. Studies on *Catharanthus roseus* have shown that oxidative stress elicits monoterpene indole alkaloids production, which has roles in antioxidant defense [66]. Indole phytoalexin accumulates in *Brassica* species with hyper-resistance to microbial infection [67]. However, indole alkaloids are only produced in a few plants, such as *Catharanthus roseus* [68], *Rauwolfia serpentina* [69], *Aspidosperma* [70], and *Camptotheca acuminate* [71]. Typically, only small amounts of indole alkaloids are found in plants with the exception of *Catharanthus roseus,* which is the sole source in the commercial production of various indole alkaloids [6]. In this study, the indole alkaloid (6-hydroxyl-1H-indol-3-yl) carboxylic acid methyl ester increased (Fig. 1b) with the accumulation of indole and tryptophan in *C. terniflora* leaves under high level of UV-B irradiation followed by an incubation in the dark (Fig. 6), suggesting that the induction of indole alkaloids occurs via activation of indole/tryptophan pathway (Fig. 8).

Methylation modification of secondary metabolites, such as indole derivatives, is important for the regulation of plant development [72]. Methylation usually occurs via the donation of a universal methyl group from S-adenosyl-L-methionine, which is catalyzed from methionine by metK [37]. In this study, metK increased in *C. terniflora* leaves after exposure to high level of UV-B irradiation and the dark (Fig. 4), indicating that the plant’s defense is enhanced through the methylation of indole alkaloids.

## Conclusions

The metabolic process of indole alkaloids in *Clematis terniflora* DC. leaves after exposure to high level of UV-B irradiation and the dark was investigated on both proteomic and metabolomic levels. Proteomic and metabolomic analyses revealed a decrease in photosynthesis and carbohydrate metabolism, respectively. By contrast, amino acid metabolism was activated by the binary stress. Additionally, L-SD activity was enhanced after binary stress, implying that the increase in amino acid metabolism contributed to an enhancement of shikimate metabolic pathway. The genes participated in the metabolic process from shikimate to L-tryptophan were concurrently upregulated by the binary stress. Metabolites involved in indole biosynthesis (shikimate metabolic) pathway including anthranilate, indole, and L-tryptophan, increased by the binary stress, as well as the enzymatic activity of L-tryptophan synthase. This suggests an enhancement in shikimate metabolic pathway promotes the indole/tryptophan pathway. Therefore, it can be concluded that the metabolic process to indole alkaloids in *Clematis terniflora* DC. leaves are enhanced after exposure to high level of UV-B irradiation and the dark. Future studies are necessary to examine any variation in downstream indole alkaloids.

## Additional files

Additional file 1: Table S1. List of primers used for qRT-PCR experiments. (DOCX 22 kb)
Additional file 2: Table S2. List of proteins identified in *Clematis terniflora* DC. leaves after exposure to high level of UV-B irradiation for 5 h and the dark for 36 h. (DOCX 36 kb)
Additional file 3: Table S3. List of metabolites identified in *Clematis terniflora* DC. leaves after exposure to high level of UV-B irradiation for 5 h and the dark for 36 h. (DOCX 51 kb)

## Abbreviations

2-DE: Two-dimensional chain electrophoresis
AAAs: Aromatic amino acids
AS: Anthranilate synthase
CHAPS: 3-[(3-cholamidopropyl) dimethylammonio]-1-propanesulfonate
CHO: Carbohydrate
CM: Chorismate mutase
CS: Chorismate synthase
DHAPS: 3-deoxy-D-arabino-heptulosonate 7-phosphate synthase
DTT: Dithiothreitol
EPSPS: 5-enolpyruvylshikimate-3-phosphate synthase
GAPDH: Glyceraldehyde-3-phosphate dehydrogenase
GC-TOF-MS: Gas chromatograph time-of-flight mass spectrometry
HPLC-TOF-MS/MS: High performance liquid chromatography time-of-flight mass spectrometry
IAA: Indole-3-acetic acid
IEF: Isoelectric focusing
IGPS: Indole-3-glycerol phosphate synthase
IPG: Immobilized pH gradient
KEGG: Kyoto Encyclopedia of Genes and Genomes
L-SD: L-serine deaminase
MALDI-TOF-MS/MS: Matrix-assisted laser desorption ionization time-of-flight mass spectrometry
metH: 5-Methyltetrahydropteroyltriglutamate-homocysteine methyltransferase
metK: S-adenosyl-L-methionine synthetase
PAI: Phosphoribosylanthranilate isomerase
PAL: Phenylalanine ammonia-lyase
PAT: Phosphoribosylanthranilate transferase
Phe: L-phenylalanine
qRT-PCR: Quantitative reverse transcription polymerase chain reaction
SK: Shikimate kinase
TCA: Tricarboxylic acid
Trp: L-tryptophan
TS: Tryptophan synthase
Tyr: L-tyrosine
UV-B: Ultraviolet-B
