# Supplementary material for: Enhanced metabolic process to indole alkaloids in Clematis terniflora DC. after exposure to high level of UV-B irradiation followed by the dark
Source: BMC Plant Biol. 2016 Oct 24;16:231. doi: 10.1186/s12870-016-0920-3 (PMC5078895; doi:10.1186/s12870-016-0920-3)
Supplement: Additional file 1: Table S1. — List of primers used for qRT-PCR experiments. (DOCX 22 kb) [file 12870_2016_920_MOESM1_ESM.docx]

**Table S1. List of primers used for qRT-PCR experiments**

| **Gene** | **Forward primer (5' to 3')** | **Reverse primer (5' to 3')** |
| --- | --- | --- |
| *CtGAPDH* | AACCCTGAGGAGATTCCA | CACCACCCTTCAAGTGAGCAG |
| *CtSK* | AGCTTGCGTGCAATCCG | TCTGGCTAGACGTGCCTTT |
| *CtEPSPS* | TTTACACGGACAGGAGGACA | TGGGAAATGCGGGAACT |
| *CtCS* | GCCACAGCCATGATGATACA | GTGCCAATGGTGGAAGC |
| *CtAS* | CATGCACCACGCCGTAA | AGCGAGGAGAAACCCAAA |
| *CtPAT* | CGGAGCAACAAACAGAAGAC | GCAAGAATAGAAGCCCCAGT |
| *CtPAI* | GGGATCGTGCTAGAACTGC | GCCACCCTTCTTTGCTTC |
| *CtIGPS* | AGCGTCCCTTGCTTTGC | CGGTCCATTTCCCTTTCATC |
| *CtTS* | TAAGGTTGATGGCGATGG | GCGCAGCAGGGTGTTAA |
