# Supplementary material for: Enhanced metabolic process to indole alkaloids in Clematis terniflora DC. after exposure to high level of UV-B irradiation followed by the dark
Source: BMC Plant Biol. 2016 Oct 24;16:231. doi: 10.1186/s12870-016-0920-3 (PMC5078895; doi:10.1186/s12870-016-0920-3)
Supplement: Additional file 2: Table S2. — List of proteins identified in Clematis terniflora DC. leaves after exposure to high level of UV-B irradiation for 5 h and the dark for 36 h. (DOCX 36 kb) [file 12870_2016_920_MOESM2_ESM.docx]

Table S2. List of proteins identified in *Clematis terniflora* DC. leaves after exposure to high level of UV-B irradiation for 5 h and the dark for 36 h

| no. | accession no. ^a^ | description | species | score ^b^ | ratio ^c^ | function^d^ |
| --- | --- | --- | --- | --- | --- | --- |
| 1 | gi\|8134570 | 5-methyltetrahydropteroyltriglutamate--homocysteine methyltransferase | *Catharanthus roseus (Madagascar periwinkle)* | 123 | 1.787±0.065 | amino acid metabolism |
| 2 | gi\|145049666 | S-adenosyl-L-methionine synthetase | *Ipomoea batatas (sweet potato)* | 168 | 1.627±0.113 | amino acid metabolism |
| 3 | gi\|75309266 | S-adenosylmethionine synthase 2 | *Suaeda salsa* | 482 | 9.108±0.797 | amino acid metabolism |
| 4 | gi\|297737594 | Unnamed protein product [Rare lipoprotein A (RlpA)-like double-psi beta-barrel] | *Vitis vinifera* | 47 | 4.815±0.321 | cell wall |
| 5 | gi\|242087501 | Hypothetical protein SORBIDRAFT_09g014711 | *Sorghum bicolor (sorghum)* | 212 | 3.833±0.289 | cell |
| 6 | gi\|242087501 | Hypothetical protein SORBIDRAFT_09g014711 | *Sorghum bicolor (sorghum)* | 51 | 3.706±0.212 | cell |
| 7 | gi\|255585293 | Late embryogenesis abundant protein Lea14-A, putative | *Ricinus communis (castor bean)* | 142 | 1.792±0.166 | development |
| 8 | gi\|169211 | Ethylene-forming enzyme | *Petunia x hybrida* | 82 | 1.479±0.073 | hormone metabolism |
| 9 | gi\|158325159 | Acireductone dioxy genase | *Solanum tuberosum (potato)* | 214 | 1.809±0.035 | metal handling |
| 10 | gi\|18424166 | Ftsh9 (FtsH protease 9) | *Arabidopsis thaliana (thale cress)* | 43 | 17.362±1.360 | protein |
| 11 | gi\|147865125 | Hypothetical protein (Ubiquitin ligase complex) | *Vitis vinifera* | 44 | 1.187±0.076 | protein |
| 12 | gi\|308813083 | Integral membrane family protein | *Ostreococcus tauri* | 47 | 18.105±1.713 | protein |
| 13 | gi\|168175 | Ribulose-1,5-bisphosphate carboxylase/oxygenase large subunit | *Fuchsia cyrtandroides* | 74 | 4.298±0.258 | photosynthesis |
| 14 | gi\|115780 | Chlorophyll a-b binding protein, chloroplastic | *Spinacia oleracea (spinach)* | 214 | 1.720±0.196 | photosynthesis |
| 15 | gi\|115778 | Chlorophyll a-b binding protein 1, chloroplastic | *Sinapis alba (white mustard)* | 154 | 3.142±0.151 | photosynthesis |
| 16 | gi\|115781 | Chlorophyll a-b binding protein 16, chloroplastic | *Nicotiana tabacum (common tobacco)* | 288 | 2.255±0.263 | photosynthesis |
| 17 | gi\|464775 | Superoxide dismutase [Mn], mitochondrial | *Hevea brasiliensis* | 240 | 1.290±0.055 | redox |
| 18 | gi\|134598 | Superoxide dismutase [Cu-Zn] 4AP | *Zea mays* | 252 | 1.500±0.121 | redox |
| 19 | gi\|2695711 | Cytochome b5 | *Olea europaea (common olive)* | 213 | 1.267±0.029 | redox |
| 20 | gi\|115471157 | Os07g0212200 (MRNA-binding protein) | *Oryza sativa Japonica Group* | 52 | 2.241±0.251 | RNA |
| 21 | gi\|115471157 | Os07g0212200 (MRNA-binding protein) | *Oryza sativa Japonica Group* | 240 | 3.213±0.177 | RNA |
| 22 | gi\|133246 | 28 kDa ribonucleoprotein, chloroplastic | *Nicotiana sylvestris (wood tobacco)* | 103 | 1.317±0.016 | RNA |
| 23 | gi\|224075860 | Predicted protein (Cinnamyl alcohol dehydrogenases) | *Populus trichocarpa (Populus balsamifera subsp. trichocarpa)* | 56 | 2.252±0.268 | secondary metabolism |
| 24 | gi\|75329266 | Probable cinnamyl alcohol dehydrogenase 1 | *Oryza sativa Japonica Group* | 119 | 1.659±0.138 | secondary metabolism |
| 25 | gi\|147834991 | Hypothetical protein (Phenylalanine ammonia-lyase) | *Vitis vinifera* | 47 | 2.626±0.141 | secondary metabolism |
| 26 | gi\|37704431 | Cytosolic class I small heat shock protein 6 | *Nicotiana tabacum (common tobacco)* | 63 | 1.592±0.200 | stress |
| 27 | gi\|116329 | Endochitinase A | *Zea mays* | 112 | 14.659±0.753 | stress |
| 28 | gi\|255553131 | Disease resistance protein RPS2, putative | *Ricinus communis (castor bean)* | 48 | 1.778±0.069 | stress |
| 29 | gi\|25282627 | Hypothetical protein F16N3.7 (Cytochrome P450 homology) | *Arabidopsis thaliana (thale cress)* | 51 | 3.487±0.305 | miscellaneous |
| 30 | gi\|111072018 | Peroxidase 1 | *Catharanthus roseus (Madagascar periwinkle)* | 112 | 1.306±0.116 | miscellaneous |
| 31 | gi\|297791425 | Late embryogenesis abundant domain-containing protein | *Arabidopsis lyrata subsp. lyrata* | 47 | 1.566±0.132 | not assigned |
| 32 | gi\|224075114 | Predicted protein | *Populus trichocarpa (Populus balsamifera subsp. trichocarpa)* | 156 | 1.279±0.041 | not assigned |
| 33 | gi\|224122216 | Predicted protein (Demethylmenaquinone methyltransferase) | *Populus trichocarpa (Populus balsamifera subsp. trichocarpa)* | 62 | 0.657±0.065 | C1-metabolism |
| 34 | gi\|262410515 | Triosphosphate isomerase-like protein type II | *Dimocarpus longan* | 289 | 0.628±0.022 | glycolysis |
| 35 | gi\|1351137 | Sucrose synthase | *Solanum tuberosum (potato)* | 44 | 0.588±0.015 | major CHO metabolism |
| 36 | gi\|1351137 | Sucrose synthase | *Solanum tuberosum (potato)* | 47 | 0.579±0.057 | major CHO metabolism |
| 37 | gi\|255582813 | Conserved hypothetical protein | *Ricinus communis (castor bean)* | 95 | 0.766±0.034 | mitochondrial electron transport |
| 38 | gi\|3122572 | NADH-ubiquinone oxidoreductase 75 kDa subunit, mitochondrial | *Solanum tuberosum (potato)* | 104 | 0.499±0.056 | mitochondrial electron transport |
| 39 | gi\|45477151 | Nucleoside diphosphate kinase 4, chloroplastic | *Spinacia oleracea (spinach)* | 345 | 0.663±0.052 | nucleotide metabolism |
| 40 | gi\|399213 | ATP-dependent Clp protease ATP-binding subunit clpA homolog CD4B, chloroplastic | *Solanum lycopersicum (Lycopersicon esculentum)* | 234 | 0.590±0.068 | protein |
| 41 | gi\|224109060 | Predicted protein (Phosphoglycerate kinase) | *Populus trichocarpa (Populus balsamifera subsp. trichocarpa)* | 871 | 0.749±0.037 | photosynthesis |
| 42 | gi\|295292099 | Ribulose-1,5-bisphosphate carboxylase/oxygenase large subunit | *Berberis asiatica* | 46 | 0.492±0.049 | photosynthesis |
| 43 | gi\|63103251 | Ribulose-1,5-bisphosphate carboxylase/oxygenase large subunit | *Pulsatilla cernua* | 697 | 0.525±0.093 | photosynthesis |
| 44 | gi\|9967892 | Ribulose bisphosphate carboxylase oxygenase | *Doerpfeldia cubensis* | 165 | 0.716±0.026 | photosynthesis |
| 45 | gi\|194400654 | Ribulose-1,5-bisphosphate carboxylase/oxygenase large subunit | *Campanula grossheimii* | 576 | 0.492±0.047 | photosynthesis |
| 46 | gi\|241992453 | Ribulose-1,5-bisphosphate carboxylase/oxygenase large subunit | *Clematis armandii* | 569 | 0.546±0.059 | photosynthesis |
| 47 | gi\|115786 | Chlorophyll a-b binding protein, chloroplastic | *Zea mays* | 270 | 0.667±0.060 | photosynthesis |
| 48 | gi\|559005 | Ascorbate peroxidase | *Nicotiana tabacum (common tobacco)* | 160 | 0.653±0.020 | redox |
| 49 | gi\|255566959 | NADH-cytochrome B5 reductase, putative | *Ricinus communis (castor bean)* | 160 | 0.311±0.030 | redox |
| 50 | gi\|13310811 | Ankyrin-repeat protein HBP1 | *Nicotiana tabacum (common tobacco)* | 92 | 0.731±0.050 | RNA |
| 51 | gi\|123548 | 17.8 kDa class I heat shock protein | *Daucus carota (carrot)* | 85 | 0.272±0.027 | stress |
| 52 | gi\|2347090 | Low molecular weight heat shock protein PvHSP17-19 | *Phaseolus vulgaris* | 74 | 0.382±0.024 | stress |
| 53 | gi\|115472 | Carbonic anhydrase, chloroplastic | *Spinacia oleracea (spinach)* | 126 | 0.647±0.028 | TCA |

^a^ accession no., the MASCOT results of MALDI-TOF-MS/ MS searched from the NCBInr database.

^b^ scores, protein scores are derived from ions scores as a non-probabilistic basis for ranking protein hits in the Mascot Search Results, individual ions scores >43 indicate identity or extensive homology (*P*<0.05).

^c^ ratio, dividing mean value of post-treatment (exposure to high level of UV-B irradiation followed by the dark) by mean value of pre-treatment. If the ratio>1, it means increase; If the ratio<1, it means decrease.

^d^ function, protein function categorized using MapMan bin codes.

C1, one-carbon; CHO, carbohydrate; TCA, tricarboxylic acid.
