# Supplementary material for: Enhanced metabolic process to indole alkaloids in Clematis terniflora DC. after exposure to high level of UV-B irradiation followed by the dark
Source: BMC Plant Biol. 2016 Oct 24;16:231. doi: 10.1186/s12870-016-0920-3 (PMC5078895; doi:10.1186/s12870-016-0920-3)
Supplement: Additional file 3: Table S3. — List of metabolites identified in Clematis terniflora DC. leaves after exposure to high level of UV-B irradiation for 5 h and the dark for 36 h. (DOCX 51 kb) [file 12870_2016_920_MOESM3_ESM.docx]

Table S3. List of metabolites identified in *Clematis terniflora* DC. leaved after exposure to high level of UV-B irradiation for 5 h and the dark for 36 h

| no. | name | similarity^a^ | retention time | UV-B + dark^b^ | Pre-treatment^c^ | fold-change^d^ | class^e^ |
| --- | --- | --- | --- | --- | --- | --- | --- |
| 1 | O-Acetyl-L-serine | 322.5333 | 11.8749,0 | 0.000744084 | 3.92365E-05 | 18.96409645 | Amino acids |
| 2 | Serine | 597.1111 | 11.3165,0 | 0.007158851 | 0.002010969 | 3.559901885 | Amino acids |
| 3 | Homoserine | 201 | 15.1061,0 | 0.000284932 | 3.74033E-05 | 7.617822212 | Amino acids |
| 4 | N-Carbamyl-L-glutamate | 230.8889 | 16.6701,0 | 7.65835E-05 | 8.62559E-06 | 8.878632728 | Amino acids |
| 5 | Leucine | 793.5385 | 10.1066,0 | 0.005050605 | 3.96745E-05 | 127.3009617 | Amino acids |
| 6 | Acetanilide | 508.2778 | 10.2609,0 | 0.001411381 | 0.000427036 | 3.305065679 | Benzenoids |
| 7 | Sedoheptulose | 361.3 | 17.5414,0 | 0.002742378 | 0.001534803 | 1.786795228 | Carbohydrates |
| 8 | Melibiose | 496 | 25.2432,0 | 0.010593082 | 0.003626104 | 2.921339695 | Carbohydrates |
| 9 | Arbutin | 372.4 | 23.0366,0 | 0.045930349 | 0.014250712 | 3.223021247 | Carbohydrates |
| 10 | 1,5-Anhydroglucitol | 498.0588 | 16.7234,0 | 0.025184751 | 0.010813254 | 2.329063066 | Carbohydrates |
| 11 | Lactulose | 542.5714 | 23.8965,0 | 0.000243542 | 0.000150582 | 1.617335142 | Carbohydrates |
| 12 | Gentiobiose | 730.6875 | 24.7758,0 | 0.03028112 | 0.016166614 | 1.873065069 | Carbohydrates |
| 13 | Prunin | 430.5 | 25.2968,0 | 0.00082719 | 0.000537896 | 1.537824495 | Flavonoids |
| 14 | Palmitoleic acid | 388.8333 | 18.6397,0 | 0.0164987 | 0.001183978 | 13.93497432 | Lipids |
| 15 | Adenosine | 439.6429 | 23.5581,0 | 0.001068506 | 0.000257194 | 4.154473826 | Nucleosides |
| 16 | Purine riboside | 486 | 21.5383,0 | 0.000304326 | 0.000122688 | 2.480477293 | Nucleosides |
| 17 | Mucic acid | 502.75 | 18.5186,0 | 7.87741E-05 | 1.16038E-05 | 6.788641517 | Organic acids |
| 18 | 4-Hydroxybutyrate | 543.75 | 9.68545,0 | 0.00223173 | 9.5464E-09 | 233777.0747 | Organic acids |
| 19 | Succinic acid | 334.5 | 10.822,0 | 0.0013162 | 9.5464E-09 | 137873.9598 | Organic acids |
| 20 | 2,3-Dimethylsuccinic acid | 572.9375 | 11.3913,0 | 0.001832411 | 0.000971172 | 1.886804683 | Organic acids |
| 21 | Estra-1,3,5(10)-triene-3,6beta,17beta-triol triacetate | 370.875 | 24.8303,0 | 0.000209192 | 0.000102534 | 2.040218459 | Steroids |
| 22 | Pyridoxal phosphate | 250 | 21.4324,0 | 0.000119091 | 3.44172E-05 | 3.460205721 | Vitamins and Cofactors |
| 23 | Cyclohexane-1,2-diol | 364.75 | 10.1914,0 | 0.000877047 | 0.000226002 | 3.880703355 | Not assigned |
| 24 | D-Glycerol 1-phosphate | 502 | 15.7713,0 | 0.001600407 | 0.000632095 | 2.531906718 | Not assigned |
| 25 | Methyl Phosphate | 759.8667 | 8.7899,0 | 0.001223999 | 0.000648689 | 1.886881191 | Not assigned |
| 26 | Dihydroxyacetone | 427.6364 | 9.7957,0 | 0.003765288 | 0.00022902 | 16.44085162 | Not assigned |
| 27 | Creatine | 475.5556 | 14.6873,0 | 0.00285348 | 0.004336767 | 0.657974042 | Amino acids |
| 28 | N-Ethylglycine | 538.0667 | 11.9726,0 | 0.001371756 | 0.003115276 | 0.440331971 | Amino acids |
| 29 | Allose | 535.7222 | 14.7919,0 | 8.54999E-09 | 0.003672627 | 2.32803E-06 | Carbohydrates |
| 30 | Galactonic acid | 818.5 | 18.0746,0 | 0.00035372 | 0.002503742 | 0.14127654 | Carbohydrates |
| 31 | Sucrose | 800 | 23.4639,0 | 8.54999E-09 | 0.008858031 | 9.65224E-07 | Carbohydrates |
| 32 | Lyxose | 894 | 14.6241,0 | 8.54999E-09 | 0.004369833 | 1.95659E-06 | Carbohydrates |
| 33 | D-Arabitol | 883.2778 | 15.3435,0 | 0.004595781 | 0.009460981 | 0.485761579 | Carbohydrates |
| 34 | Xylitol | 534.4375 | 15.4208,0 | 0.002768318 | 0.004453516 | 0.621602773 | Carbohydrates |
| 35 | Glucuronic acid | 558.2308 | 17.7101,0 | 8.54999E-09 | 0.001138126 | 7.51234E-06 | Carbohydrates |
| 36 | Gluconic acid | 909.1667 | 18.1229,0 | 8.54999E-09 | 0.005730715 | 1.49196E-06 | Carbohydrates |
| 37 | D-Ribose | 665.5 | 14.9542,0 | 0.000806854 | 0.001652903 | 0.488143725 | Carbohydrates |
| 38 | Glucose 6-phosphate | 685.6364 | 20.9387,0 | 0.000278456 | 0.000467821 | 0.595219429 | Carbohydrates |
| 39 | Maltose | 740.9286 | 24.4122,0 | 0.000158822 | 0.000301291 | 0.527140477 | Carbohydrates |
| 40 | D-Digitoxose | 371.5385 | 14.3017,0 | 0.000764404 | 0.001550942 | 0.492864066 | Carbohydrates |
| 41 | D-Ribonate | 592.3333 | 15.1468,0 | 8.54999E-09 | 0.001385106 | 6.17281E-06 | Carbohydrates |
| 42 | 6-Deoxy-D-glucose | 218.3636 | 15.473,0 | 0.00034746 | 0.000659165 | 0.527120455 | Carbohydrates |
| 43 | 3,6-Anhydro-D-galactose | 623.0556 | 15.7196,0 | 0.0033912 | 0.006746694 | 0.502646129 | Carbohydrates |
| 44 | 2-Deoxy-D-galactose | 629.5833 | 16.2351,0 | 8.54999E-09 | 0.000228599 | 3.74017E-05 | Carbohydrates |
| 45 | Tricetin | 425.0909 | 28.6522,0 | 0.000168412 | 0.000285944 | 0.588969822 | Flavonoids |
| 46 | 4-Hydroxy-6-methyl-2-pyrone | 426.4 | 13.0278,0 | 8.54999E-09 | 0.003026352 | 2.82518E-06 | Latctones |
| 47 | Linolenic acid | 717.0556 | 20.239,0 | 8.54999E-09 | 0.00382858 | 2.2332E-06 | Lipids |
| 48 | Palmitic acid | 963.6111 | 18.6973,0 | 8.54999E-09 | 0.024770493 | 3.45168E-07 | Lipids |
| 49 | Linoleic acid | 746.5 | 20.1751,0 | 8.54999E-09 | 0.000578467 | 1.47804E-05 | Lipids |
| 50 | Prostaglandin A2 | 458.2353 | 23.9894,0 | 0.000263974 | 0.000406019 | 0.650152364 | Lipids |
| 51 | Linoleic acid methyl ester | 608.7692 | 19.1951,0 | 0.000114524 | 0.000311621 | 0.367510614 | Lipids |
| 52 | Uridine | 552.9444 | 22.3082,0 | 0.02154124 | 0.034295301 | 0.628110548 | Nucleosides |
| 53 | 5,6-Dihydrouracil | 379.8182 | 13.1371,0 | 8.54999E-09 | 0.001274229 | 6.70993E-06 | Nuclesides |
| 54 | Maleic acid | 592.2 | 10.5786,0 | 2.92111E-05 | 0.000359131 | 0.081338129 | Organic acids |
| 55 | Glutaric Acid | 468.9375 | 11.8177,0 | 0.001462999 | 0.003330517 | 0.439270917 | Organic acids |
| 56 | Oxalacetic acid | 468.5455 | 12.6378,0 | 8.54999E-09 | 0.026746632 | 3.19666E-07 | Organic acids |
| 57 | Glycolic acid | 812.2143 | 7.16823,0 | 8.54999E-09 | 0.000392373 | 2.17904E-05 | Organic acids |
| 58 | 2-Methylfumarate | 460.2 | 11.8095,0 | 0.021751431 | 0.046130074 | 0.471523872 | Organic acids |
| 59 | Maleamate | 552 | 13.4547,0 | 0.002574162 | 0.005660051 | 0.454794902 | Organic acids |
| 60 | 3-Isopropylbutanedioic acid | 577.8333 | 13.7216,0 | 0.002192525 | 0.007506301 | 0.292091266 | Organic acids |
| 61 | Glutaconic acid | 512.9333 | 12.4512,0 | 4.76021E-05 | 0.000190498 | 0.249882259 | Organic acids |
| 62 | Aconitic Acid | 208.375 | 15.681,0 | 3.86628E-05 | 6.33514E-05 | 0.610290569 | Organic acids |
| 63 | N-ethylmaleamic acid | 404.2222 | 12.7518,0 | 0.014052545 | 0.025636175 | 0.548152957 | Organic acids |
| 64 | Heptadecanoic acid | 674.4286 | 19.5864,0 | 0.000112016 | 0.000314258 | 0.356446226 | Organic acids |
| 65 | Estrone | 248.9 | 24.4304,0 | 0.000157792 | 0.000351428 | 0.449003496 | Steroids |
| 66 | Squalene | 437 | 25.1754,0 | 0.00149115 | 0.002949632 | 0.505537699 | Terpenoids |
| 67 | Xanthine | 283.5 | 18.5048,0 | 6.17129E-06 | 6.92395E-05 | 0.089129574 | Not assigned |
| 68 | Spermidine | 257.4286 | 20.1573,0 | 9.02667E-05 | 0.000245434 | 0.367784263 | Not assigned |
| 69 | Acetol | 467.7143 | 13.3657,0 | 8.54999E-09 | 8.988E-05 | 9.51267E-05 | Not assigned |
| 70 | Threitol | 760.2222 | 12.9487,0 | 0.005851936 | 0.011908943 | 0.49139007 | Not assigned |
| 71 | Glutaraldehyde | 271.3333 | 10.4449,0 | 0.000126657 | 0.000517304 | 0.244840698 | Not assigned |
| 72 | DL-dihydrosphingosine | 556.3846 | 22.5357,0 | 3.06803E-06 | 0.000263505 | 0.011643172 | Not assigned |
| 73 | 2-Monopalmitin | 250 | 22.8555,0 | 0.000679914 | 0.001138324 | 0.597293506 | Not assigned |
| 74 | 6-Methylprevitamin D | 262.1111 | 28.1226,0 | 7.68297E-05 | 0.000159788 | 0.480823305 | Not assigned |
| 75 | Valine | 424.2222 | 7.58178,0 | 0.002155784 | 0.002548228 | 0.845993354 |  |
| 76 | Alanine | 608.1667 | 7.67344,0 | 0.002020582 | 0.002173904 | 0.92947161 |  |
| 77 | Hydroxylamine | 412.1667 | 8.02,0 | 0.000650617 | 0.000781112 | 0.832937784 |  |
| 78 | Oxalic acid | 641.2778 | 8.03928,0 | 0.050962445 | 0.058186169 | 0.875851527 |  |
| 79 | 3-Hydroxybutyric acid | 332 | 8.53197,0 | 0.008784336 | 0.008969163 | 0.979393135 |  |
| 80 | Malonic acid | 536.1111 | 9.13191,0 | 0.000493583 | 0.000533856 | 0.92456244 |  |
| 81 | 3,4-Dihydroxycinnamic acid | 899.4444 | 19.5198,0 | 0.009168768 | 0.013322737 | 0.688204524 |  |
| 82 | Norleucine | 529.75 | 9.36066,0 | 0.003206533 | 0.003771441 | 0.850214045 |  |
| 83 | Methylmalonic acid | 623.1667 | 9.37998,0 | 0.002205356 | 0.001919604 | 1.148859814 |  |
| 84 | Hydroxyurea | 412.25 | 9.57879,0 | 0.000332566 | 0.000360992 | 0.92125689 |  |
| 85 | Pyrophosphate | 283.125 | 9.83625,0 | 0.049364996 | 0.042747519 | 1.15480377 |  |
| 86 | Citraconic acid | 509.8333 | 11.3357,0 | 0.000350186 | 0.000505998 | 0.692070268 |  |
| 87 | Tartronic acid | 377.4167 | 11.5184,0 | 0.000292375 | 0.000286774 | 1.019532236 |  |
| 88 | L-Allothreonine | 296.8 | 11.706,0 | 0.000234303 | 0.000276269 | 0.848096668 |  |
| 89 | 3-Methylglutaric Acid | 288.8889 | 12.0769,0 | 0.009163626 | 0.007254739 | 1.263122756 |  |
| 90 | D-erythronolactone | 570.5 | 12.1728,0 | 0.009196303 | 0.00993879 | 0.925294035 |  |
| 91 | Beta-Alanine | 475.6667 | 12.3072,0 | 0.001595525 | 0.001786857 | 0.892922492 |  |
| 92 | L-Malic acid | 555.5 | 12.8245,0 | 0.000546368 | 0.000452442 | 1.207598258 |  |
| 93 | L-Threose | 617.5 | 12.5309,0 | 0.000106928 | 0.000121442 | 0.880490096 |  |
| 94 | Capric Acid | 249 | 12.5759,0 | 0.0004441 | 0.000453954 | 0.978293556 |  |
| 95 | Bis(2-hydroxypropyl)amine | 682.1111 | 13.0182,0 | 0.005007199 | 0.005789215 | 0.864918587 |  |
| 96 | 2-Deoxyerythritol | 517.1 | 10.227,0 | 0.001204044 | 0.0018007 | 0.668653257 |  |
| 97 | 3-Hexenedioic acid | 570.5714 | 13.1147,0 | 0.005892153 | 0.006987154 | 0.843283724 |  |
| 98 | 4-Hydroxyquinazoline | 466.1 | 13.5114,0 | 0.003931965 | 0.004288986 | 0.916758693 |  |
| 99 | 3-Hydroxypropionic acid | 464.375 | 13.9193,0 | 0.002939089 | 0.003267743 | 0.899424767 |  |
| 100 | 3-Phenyllactic acid | 381.75 | 13.99,0 | 0.001085607 | 0.001208836 | 0.898059727 |  |
| 101 | Beta-Glutamic acid | 396.2222 | 14.1994,0 | 0.000659664 | 0.000651162 | 1.013056384 |  |
| 102 | Thymidine | 481.8 | 14.42,0 | 0.003167278 | 0.0030305 | 1.045133724 |  |
| 103 | 5-Aminovaleric acid | 261.1429 | 15.4107,0 | 8.87609E-05 | 8.66965E-05 | 1.023811913 |  |
| 104 | Ribitol | 667.7647 | 15.4872,0 | 0.003928504 | 0.003476941 | 1.129873517 |  |
| 105 | Diglycerol | 723.5625 | 15.74,0 | 0.001851158 | 0.002713044 | 0.682317818 |  |
| 106 | Glucose-1-phosphate | 910.1111 | 15.8023,0 | 0.116042949 | 0.136256403 | 0.851651348 |  |
| 107 | 2-Deoxy-D-glucose | 594.5625 | 16.0212,0 | 0.07706965 | 0.095256968 | 0.809070997 |  |
| 108 | Citric acid | 935 | 16.3472,0 | 0.277287109 | 0.218432342 | 1.269441635 |  |
| 109 | Alpha-D-glucosamine 1-phosphate | 544.6364 | 16.4929,0 | 0.000283866 | 0.000221351 | 1.282425726 |  |
| 110 | N-Acetyl-L-phenylalanine | 444.6667 | 16.5006,0 | 0.000184833 | 0.000167438 | 1.103889228 |  |
| 111 | Methyl-beta-D-galactopyranoside | 756.2222 | 16.8038,0 | 0.001657758 | 0.001825395 | 0.908163905 |  |
| 112 | Gluconic lactone | 669.8 | 17.0853,0 | 0.046240732 | 0.058458394 | 0.791002435 |  |
| 113 | Tyramine | 834.5833 | 17.4188,0 | 0.003532888 | 0.005148121 | 0.686248038 |  |
| 114 | Conduritol b epoxide | 314.0556 | 17.5233,0 | 0.002748776 | 0.00343915 | 0.799260348 |  |
| 115 | Isopropyl-beta-D-thiogalactopyranoside | 477 | 18.7776,0 | 0.000940916 | 0.001096515 | 0.858096408 |  |
| 116 | Carbobenzyloxy-L-leucine | 212.3333 | 18.8455,0 | 2.61221E-05 | 2.86121E-05 | 0.912973881 |  |
| 117 | Myo-inositol | 622.1667 | 19.0172,0 | 0.02405641 | 0.023579354 | 1.020231921 |  |
| 118 | N-Acetyl-beta-D-mannosamine | 765.3889 | 19.1437,0 | 0.016224005 | 0.019800142 | 0.819388305 |  |
| 119 | Glucoheptonic acid | 627.7647 | 19.4241,0 | 0.000583301 | 0.000582151 | 1.001974149 |  |
| 120 | Cis-Phytol | 363.5 | 19.4234,0 | 0.000685022 | 0.00077311 | 0.886059799 |  |
| 121 | D-Glucoheptose | 635.1667 | 19.4137,0 | 0.000356966 | 0.000289261 | 1.234065112 |  |
| 122 | Phytol | 958.1667 | 19.8037,0 | 0.176759159 | 0.154568235 | 1.143567172 |  |
| 123 | Fructose 2,6-biphosphate degr prod | 561.8333 | 20.1093,0 | 0.001011206 | 0.000837702 | 1.20711884 |  |
| 124 | Beta-Mannosylglycerate | 706.2222 | 20.2456,0 | 0.001657644 | 0.00182219 | 0.909698697 |  |
| 125 | Stearic acid | 936.9444 | 20.4425,0 | 0.005784498 | 0.00517437 | 1.117913464 |  |
| 126 | Fructose-6-phosphate | 568.5625 | 20.8708,0 | 0.000201831 | 0.000160081 | 1.260804369 |  |
| 127 | Arachidonic acid | 367 | 21.5586,0 | 0.000144996 | 0.000138803 | 1.044611306 |  |
| 128 | 1-Hydroxyanthraquinone | 330.2 | 21.7978,0 | 0.000422147 | 0.000475943 | 0.886969451 |  |
| 129 | Arachidic acid | 561.8 | 22.0644,0 | 0.007290394 | 0.009543562 | 0.763907026 |  |
| 130 | D-erythro-sphingosine | 620.8333 | 22.2581,0 | 0.06407697 | 0.052735472 | 1.215063919 |  |
| 131 | Biotin | 287.25 | 22.4895,0 | 3.03652E-05 | 3.12392E-05 | 0.972024023 |  |
| 132 | Salicin | 562 | 22.6569,0 | 0.001954588 | 0.002785052 | 0.701813873 |  |
| 133 | Cellobiose | 719.5882 | 24.0641,0 | 0.002311904 | 0.002028003 | 1.139990607 |  |
| 134 | Lactose | 725 | 24.2166,0 | 0.004747438 | 0.005292883 | 0.896947505 |  |
| 135 | Lactobionic Acid | 715.2222 | 24.4754,0 | 8.54999E-09 | 9.5464E-09 | 0.895623993 |  |
| 136 | Leucrose | 805.1667 | 25.0834,0 | 0.003255299 | 0.004730158 | 0.688200853 |  |
| 137 | Galactinol | 795.4444 | 26.1027,0 | 0.003008954 | 0.00388323 | 0.774858508 |  |
| 138 | Trehalose-6-phosphate | 441.5 | 27.3193,0 | 7.62668E-05 | 8.1865E-05 | 0.93161662 |  |
| 139 | Melezitose | 633 | 29.1489,0 | 0.002104882 | 0.001979769 | 1.063195538 |  |

^a^ simility., the LECO/Fiehn Metabolomics Library was used to identify the compound. It will give a similarity value for the compound identification accuracy. If the similarity is >700, we can think that the metabolite is reliable. If the similarity is between 200 and 700, the compound name is putative annotation.

^b^ UV-B + dark, mean value of peak area with normalization obtained from *Clematis terniflora* DC. leaved after exposure to high level of UV-B irradiation for 5 h and an incubation in the dark for 36 h.

^c^ pre-treatment, mean value of peak area with normalization obtained from *Clematis terniflora* DC. leaved pre-treatment.

^d^ fold change, dividing value of UV-B + dark by value of pre-treatment. If the fold-change value > 1, it means that metabolite is more in high level of UV-B irradiation for 5 h and an incubation in the dark for 36 h than pre-treatment. If the fold-change value >1.5 or <0.66667, it means that metabolite is in significant change.

^e^ class, metabolites were functionally categorized using the KEGG database or manually classified based on their chemical structures. Only metabolites with significant change were classified.
